# Stability of non-Newtonian nanofluid movement with heat/mass transportation passed through a hydro magnetic elongating/contracting sheet: multiple branches solutions

**DOI:** 10.1038/s41598-023-44640-3

**Published:** 2023-10-18

**Authors:** Humaira Yasmin, Azzh Saad Alshehry, Zeeshan, Abdul Hamid Ghanie, Rasool Shah

**Affiliations:** 1 Department of Basic Sciences, Preparatory Year Deanship, King Faisal University, 31982 Al-Ahsa, Saudi Arabia; 2 Department of Mathematical Sciences, Faculty of Sciences, Princess Nourah Bint Abdulrahman University, P.O. Box 84428, 11671 Riyadh, Saudi Arabia; 3 Department of Mathematics and Statistics, Bacha Khan University Charsadda, Charsadda, KP Pakistan; 4 Basic Science Department, College of Science and Theoretical Studies, Saudi Electronic University, 11673 Riyadh, Saudi Arabia; 5 Department of Mathematics, Abdul Wali Khan University Mardan, Mardan, KP Pakistan

**Keywords:** Neuroscience, Energy science and technology, Engineering, Mathematics and computing, Nanoscience and technology, Physics

## Abstract

Nanomaterials have found wide applications in many fields, leading to significant interest in the scientific world, in particular automobile thermal control, heat reservoirs, freezers, hybrid control machines, paper creation, cooling organisms, etc. The aim of the present study is to investigate the MHD non-Newtonian nanofluid and time-based stability analysis to verify the stable branch by computing the smallest eigenvalue across a slendering, extending, or shrinking sheet with thermal radiation and chemical reactions. The basic flow equations have been obtained in terms of PDEs, which are then converted to ODEs in dimensionless form via a suitable transformation. Based on the MATLAB software package bvp4c, the numerical solution has been obtained for the system of equations. A comparative study of the present and published work is impressive. The influence of evolving factors such as Prandtl number, Schmidt number, magnetic factor, heat generation/absorption, thermal, thermophoresis factor, chemical factor, second-grade fluid factor, and Brownian number on the velocities, energy, and concentration patterns is discussed through graphs. It is perceived that the temperature distribution enriches owing to the greater magnitude of the heat source. Furthermore, it is observed that a greater magnitude of radiation improves the temperature curves. It is also investigated from the present analysis that concentration and temperature profiles increase due to the growing values of the thermophoresis factor.

## Introduction

The high-intensity heat exchange criteria can no longer be met by conventional process fluids with weak conductivity because of the rising need for efficient heat transfer in different sectors. The development of modern heat exchanger performance and compactness is severely restricted by low thermal characteristics of heat transmission liquids. The thermal performance of liquids may be improved in significant ways by suspending tiny solid particles in liquids. Hence, nanofluid (NFs) may improve heat transmission and thermal properties, making them valuable. NFs unique properties make them useful in an extensive assortment of heat transfer uses. They include, but are not restricted to, automobile thermal controlling, heat reservoir, freezers, hybrid control machines, paper creation, cooling organisms, etc. In order to improve heat transmission, Choi^1^ proposed a novel fluid named as nanofluid. In order to establish a correlation between the velocities of the convectional fluid and the nanoparticles, Buongiorno^2^ introduced seven slip processes i.e., Diffusion, Brownian motion, drainage, thermophoresis, Magnus effects, diffusiophoresis, and inertia. forces. Out of these seven processes, he determined that only Brownian diffusion and thermophoresis really matter.

The proposed concept of Choi and Eastman^3^ has made it possible for researchers to find numerous ways to increase the heat transferred. Akram et al.^4^ studied the nanofluid in a vertical surface and it was concluded that due to increasing the quantity of thermophoresis and Dufour factors, the temperature and concentration of the fluid were increased. Hafeez et al.^5^ studied the Oldroyd-B fluid containing nanoparticles with radiation through a rotating channel. Ali et al.^6^ investigated the magnetized Darcy–Forchheimer model of micro-nanofluid passes through an elongated shrinking sheet. The stability analysis was also implemented in their study. Lund et al.^7^ detected the MHD influence on hybrid nanofluid enclosing viscous dissipation. Dero et al.^8^ examined the multiple solutions with stability for the nanofluid over the shrinking sheet. The slip effect was also considered at the boundary. Similarly, Dero et al.^9^ inspected the inspiration of viscous force on nanofluid flow using the shrinking sheet. Ramezanizadeh et al.^10^ portrayed the thermal characteristics effect on nanofluid flow. Akram et al.^11^ scrutinized the inspiration of thermal on 4th-grade nanofluid. A comprehensive review of NF was explained by Maleki et al.^12^. Akram et al.^13, 14^ inspected the impact of Prandtl number and thermal on magnetized couple stress nanofluid over a non-uniform channel.

The influences of magnetohydrodynamics (MHD) have gained significant consideration owing to their significant applications in industry and chemical characteristics. Numerous researchers investigated the effect of MHD in their studies. Aman et al.^15^ explored the outcome of MHD and slip factor in the stagnation region that passes through a stretching surface. Similarly, later on, the same effect of MHD was explained over the nonlinear elongated sheet along with viscous dissipation impact. Qureshi et al.^16^ investigated the morphological monolayer using hybrid NF. Rauf et al.^17^ studied the MHD hybrid nanofluid via a stretching sheet. Oreyeni et al.^18^ investigated the impact of heat source in Casson fluid with variable thermo physical phenomena. Zeeshan et al.^19^ explored the impact of MHD on buongionro nanofluid model over permeable elongated sheet with heat source. Zeeshan et al.^20^ investigated the 2D flow over permeable extended sheet with thermal and slip influence. Further, Zeeshan et al.^21^ gave the comparative analysis of nanofluid and hybrid nanofluid for heat transfer analysis over te stretched curve with melting heat influence.

Research on stretching and shrinking sheet flow plays a significant role in heat transfer that is related to industrial and manufacturing applications including the nanotechnology industry, cooling of heavy machinery, and nuclear sector technology. Cortell^22^ investigated the viscous fluid movement over stretching nonlinear surface. This study was later extended by incorporating the consequence of viscous dissipation as well as the thermal effect^23^. Next, Bachok and Ishak^24^ examined the stagnation region over the extending/shrinking sheet. A non-unique solution was obtained due to the shrinking sheet. Meanwhile, Fauzi et al.^25^ considered the shrinking/contracting sheet with a velocity slip effect in the stagnation region. Similarly, the extending/shrinking sheet problems with various physical impacts were inspected by many researchers^26–30^.

In most of the research, non-unique solutions have been obtained by many researchers, therefore, the stability analysis is essential to obtain stable branches. Merkin^31^ was the principal one who used the stability inquiry in mixed convection movement over permeable channels. Later, using this concept Weidman et al.^32^ studied a flow problem over a moving plate with stability analysis. Next, Merrill et al.^33^ studied the stagnation boundary flow over a permeable vertical sheet. The proposed model of Merrill et al.^34^ was extended by Harris et al.^35^ by incorporating the slip influence using the Brinkman flow model. Flows on stability were examined by many researchers^36–38^. They concluded that the first branch was reliable and the second was unstable.

Furthermore, heat generation/absorption is also a significant and important component in regulating heat transmission. Several manufacturing processes like hydrothermal sources, chemical reactions, nuclear power plants, and energy absorption reveal the significant applications of heat sources/sinks^39^. Hayat et al.^40^ considered the effect of heat sources/sinks with chemical processes on hybrid nanofluid through the extended sheet. The 2D flow of time-independent over the stretched sheet in the inspiration of slip and heat generation/sink was examined by Wahid et al.^41^. Li et al.^42^ studied the multiple solutions of radiated Falkner–Skan Maxwell nanofluid along with heat mass transfer across a stationary or moving wedge. Madhukesh et al.^43^ investigated the magnetised Casson nanofluid between two permeable discs with Cattaneo–Christov heat flux. Nagaraja et al.^44^ explored the impact of heat mass transfer from assisting or opposing flow with a radiation effect on ternary hybrid nanofluids through a stretching sheet. Madhukesh et al.^45^ examined the hubrid nanofluid with assisting and opposing flows over an exponentially elongated sheet. Ramesh et al.^46^ displayed the impact of a heat source on a hybrid nanofluid with thermophoresis characteristics over a nonlinear elongating sheet. Waqas et al.^47^ studied the influence of entropy, hyderothermal, and kinetic energy on nanofluid flow with solid volume friction. Similarly, Waqas et al.^48^ numerically investigated the impact of Brownian and thermophoresis on micropolar nanoparticles along with activation energy. The copper–water-based nanofluid for heat enhancement over a horizontal annulus was investigated by Waqas et al.^49^. A thermal-based investigation of the hybrid nanofluis with the Cattaneo-Christov theory was studied by Waqas et al.^50^. Shu-Bo et al.^51^ investigated the dual stratified Casson nanofluid with the effect of a magnetic dipole, generalized Fourier's, and Fick's laws. Muhammad et al.^52^ studied the hybrid nanofluid in a porous medium with a dual slip effect. Jawad et al.^53^ examined the ion slip using the 3D flow of Maxwell nanofluid through a rotating disc containing gyrotactic microorganisms. The influence of Newtonian heating, along with Fourier and Fick’s laws and a variable heat source, on MHD dusty Casson nanofluid through a stretched cylinder was investigated by Muhammad et al.^54^. Furthermore, Muhammad et al.^55^ explored the effect of chemical reaction on Maxwell nanofluid using Fourier and Fick laws over a rotating cone with thermal characteristics.

Many of the non-Newtonian fluid flow problems revealed above were examined 2D flow and have not investigated dual branches over the slendering extending/contracting sheet flow model with stability analysis. In the present analysis, an investigation is done for the 3D flow of non-Newtonian liquid over a slandering stretching/shrinking sheet enclosing the impact of chemical reaction, radiation, velocity slip factor, Brownian motion, thermophoresis factor, temperature jump, temperature and concentration jump, 2nd-grade fluid parameter, and the Schmidt number on the flow characteristics. The novelty of the current study is to investigate the multiple branches and stability analysis for the second-grade fluid over the sl3ndering stretching/shrinking sheet which has been not investigated yet. The main objectives of this investigation are.

- Firstly, an analysis of non-Newtonian second-grade fluid in three-dimensional flow is performed over the slandering extending/contracting sheet enclosing the impact of chemical processes, radiation, velocity slip factor, Brownian motion, and thermophoresis factor
- Secondly, for the nanofluid flow Buongiorno model is used
- Thirdly, multiple branches are obtained. And
- The last one is to implement a stability analysis

## Mathematical analysis

**Figure 1:**
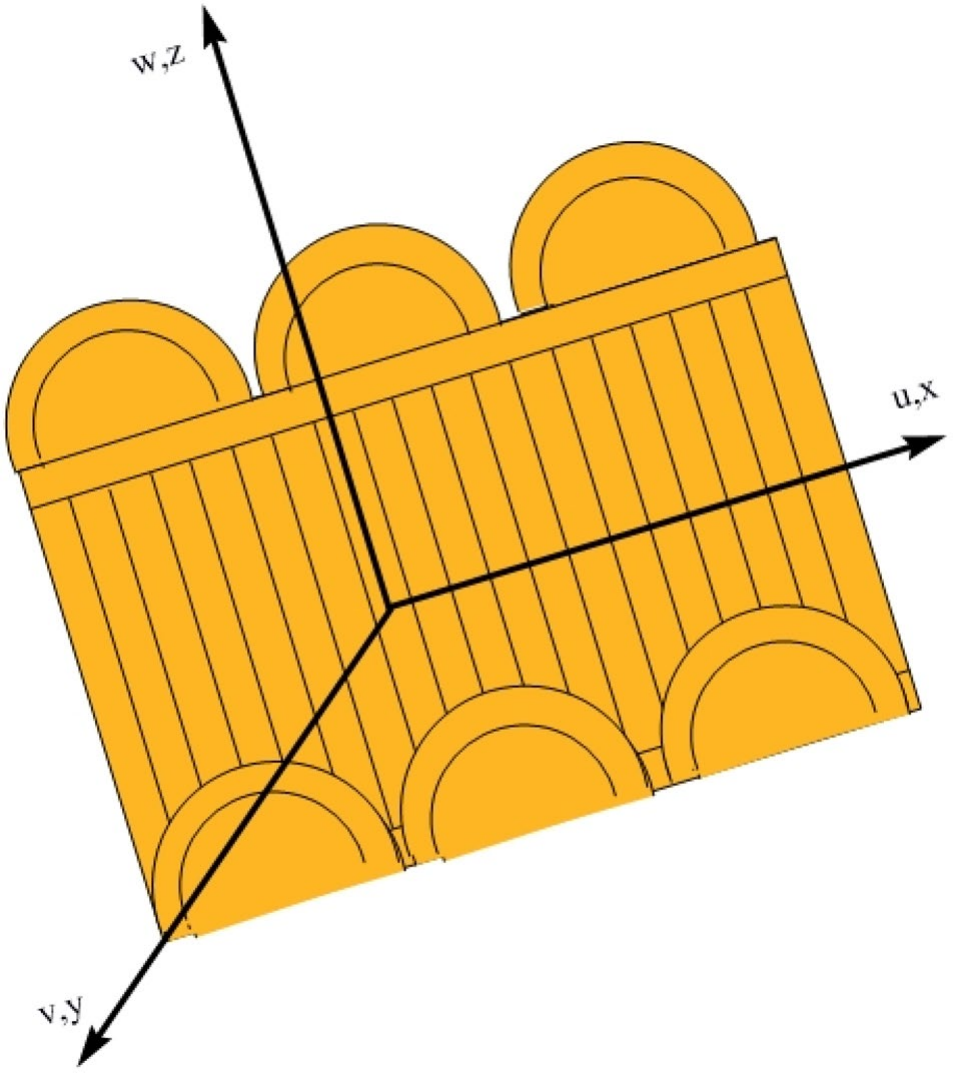
Flow over slandering stretching sheet.

Consider the magnetized non-Newtonian second-grade fluid through a stretching slendering enclosing the impacts of chemical reactions, heat generation/absorption, and radiations. The Buongiorno model is used for the present analysis to investigate the influence of nanoparticles on the flow. Figure 1 depicts the flow over the slandering stretching sheet. The sheet is stretched with constant velocity ${U}_{w}={\lambda }_{1}cx$, in the x-direction. Here, ${\lambda }_{1}$ indicates the constant stretching/shrinking parameter. It is important to notice here that ${\lambda }_{1}=0$ stands for static, ${\lambda }_{1}>0$ presents the stretching, and ${\lambda }_{1}<0$, portrays the shrinking sheet. The applied magnetic field is taken of strength ${B}_{0}$ normal to the flow.

The basic flow equations use in the current study is given below^20–24^:

1 $$\overrightarrow {\nabla \cdot } \vec{V} = 0$$

2 $$\rho \frac{{d\vec{V}}}{dt} = divS + \vec{F}$$

3 $$\frac{{d\vec{T}}}{dt} = \alpha \nabla^{2} T + \frac{1}{{\rho C_{p} }}S_{1}$$

4 $$\frac{{d\vec{C}}}{dt} = D\nabla^{2} C + S_{2}$$

The stress tensor is defined as

5 $$S = - pI + \mu A_{1} + \alpha_{1} A_{2} + \alpha_{2} A_{1}^{2} ,\vec{F} = \vec{j} \times \vec{B}$$

Here, $p$ denotes the pressure, ${\alpha }_{1}\ge 0,{\alpha }_{1}+{\alpha }_{2}=0,\mu \ge 0$ are moduli stress constant, $\overrightarrow{j}\left(\mathrm{A}/{\mathrm{m}}^{2}\right)$ is the electric current ($\overrightarrow{j}=\sigma (\overrightarrow{E}+\overrightarrow{V}\times \overrightarrow{B})$. Where ${A}_{2}$ and ${A}_{1}$ are given below

6 $$\begin{aligned} A_{1} = & \left( {{\text{grad}}\vec{V}} \right) + ({\text{grad}}\vec{V})^{T} , \\ A_{2} = & \frac{{dA_{1} }}{dt} + A_{1} \left( {{\text{grad}}\vec{V}} \right) + \left( {{\text{grad}}\vec{V}^{T} A_{1} .} \right. \\ \end{aligned}$$

Form Eqs. (5) and (6), Eq. (2) gives the velocity components in term of $u$ and $v$, respectively.

7 $$\frac{\partial x}{{\partial x}} + \frac{\partial v}{{\partial y}} + \frac{\partial w}{{\partial z}} = 0$$

8 $$\begin{aligned} \rho_{f} \left( {u\frac{\partial u}{{\partial x}} + v\frac{\partial u}{{\partial y}} + w\frac{\partial u}{{\partial z}}} \right) &= \mu \frac{{\partial^{2} u}}{{\partial z^{2} }} + \alpha_{1} \left( {u\frac{{\partial^{3} u}}{{\partial x\partial z^{2} }} - \frac{{\partial^{2} w}}{{\partial z^{2} }}\frac{\partial u}{{\partial z}} - \frac{{\partial^{2} u}}{{\partial z^{2} }}\frac{\partial u}{{\partial x}}} \right. \\ & \quad \left. { + w\frac{{\partial^{3} u}}{{\partial z^{3} }} - 2\frac{\partial u}{{\partial z}}\frac{{\partial^{2} u}}{\partial x\partial z} - 2\frac{\partial w}{{\partial z}}\frac{{\partial^{2} u}}{{\partial z^{2} }}} \right) - \sigma B^{2} \left( x \right)u \\ \end{aligned}$$

9 $$\begin{aligned} \rho_{f} \left( {u\frac{\partial v}{{\partial x}} + v\frac{\partial v}{{\partial y}} + w\frac{\partial v}{{\partial z}}} \right) &= \mu \frac{{\partial^{2} v}}{{\partial z^{2} }} + \alpha_{1} \left( {u\frac{{\partial^{3} v}}{{\partial x\partial z^{2} }} - \frac{{\partial^{2} w}}{{\partial z^{2} }}\frac{\partial v}{{\partial z}} - \frac{{\partial^{2} v}}{{\partial z^{2} }}\frac{\partial v}{{\partial x}}} \right. \\ & \quad + w\frac{{\partial^{3} v}}{{\partial z^{3} }} - 2\frac{\partial v}{{\partial z}}\frac{{\partial^{2} v}}{\partial x\partial z} - 2\frac{\partial w}{{\partial z}}\frac{{\partial^{2} v}}{{\partial z^{2} }}) - \sigma B^{2} \left( x \right)v \\ \end{aligned}$$

10 $$(\rho c)_{f} \left( {u\frac{\partial T}{{\partial x}} + v\frac{\partial T}{{\partial y}} + w\frac{\partial T}{{\partial z}}} \right) = \frac{{\partial^{2} T}}{{\partial z^{2} }} + \tau \left( {D_{B} \frac{\partial T}{{\partial z}}\frac{\partial C}{{\partial z}} + \left( {\frac{\partial T}{{\partial z}}} \right)^{2} \frac{{D_{B} }}{{T_{\infty } }}} \right) + Q_{0} \left( {T - T_{\infty } } \right) - \frac{{\partial q_{r} }}{\partial z} - q^{\prime \prime \prime }$$

11 $$u\frac{\partial C}{{\partial x}} + v\frac{\partial C}{{\partial y}} + w\frac{\partial C}{{\partial z}} = \frac{{\partial^{2} T}}{{\partial z^{2} }}\frac{{D_{T} }}{{T_{\infty } }} + D_{B} \frac{{\partial^{2} C}}{{\partial z^{2} }} - K_{1} \left( {C - C_{\infty } } \right).$$

Where ${q}^{{\prime\prime\prime}}$ is the described as

12 $$q^{\prime\prime\prime} = \frac{{kU_{w} \left( x \right)}}{{v\left( {x + y + c} \right)}}\left( {L^{*} \left( {T_{w} - T_{\infty } } \right)f^{\prime} + M^{*} \left( {T - T_{\infty } } \right)} \right.$$

In above Eq. (12),$L^{*} > 0$, $M^{*} > 0$ signifies heat generation, and $L^{*} < 0$, $M^{*} < 0$ denotes the heat absorption.

The corresponding boundary constraints are:

13 $$\begin{array}{*{20}c} {u\left( {x,y,z} \right) = \lambda_{1} U_{w} \left( x \right) + b_{1}^{*} \left( {\frac{\partial u}{{\partial z}}} \right),v\left( {x,y,z} \right) = V_{w} \left( x \right) + b_{1}^{*} \left( {\frac{\partial v}{{\partial z}}} \right),} \\ {T\left( {x,y,z} \right) = b_{2}^{*} \left( {\frac{\partial T}{{\partial z}}} \right) + T_{w} \left( x \right),{\text{C}}\left( {x,y,z} \right) = b_{3}^{*} \left( {\frac{\partial C}{{\partial z}}} \right) + C_{w} \left( x \right),} \\ {u = 0,\frac{\partial u}{{\partial z}} = 0,v = 0,\frac{\partial v}{{\partial z}} = 0,T = T_{\infty } ,C = C_{\infty } asz \to \infty .} \\ \end{array}$$

where

$b_{1}^{*} = \zeta_{1} \left( {\frac{{2 - f_{1} }}{{f_{1} }}} \right)(x + y + c)^{{\frac{1 - n}{2}}} ,$ and $K_{B}$ is Boltzmann constant and $d$ is particle diameter.

14 $$\begin{aligned} b_{2}^{*} = & \zeta_{2} \left( {\frac{2 - b}{b}} \right)(x + y + c)^{{\frac{1 - n}{2}}} ,\zeta_{2} = \frac{{\zeta_{1} }}{{{\text{Pr}}}}\left( {\frac{2\gamma}{{\gamma + 1}}} \right) \\ b_{3}^{*} = & \zeta_{3} \left( {\frac{2 - m}{m}} \right)(x + y + c)^{{\frac{1 - \pi }{2}}} ,\zeta_{3} = \left( {\frac{2\gamma }{{\gamma + 1}}} \right)\frac{{\zeta_{2} }}{Pr} \\ \end{aligned}$$

$\zeta_{1} = \frac{{K_{B} T}}{{\sqrt 2 \pi^{2} p^{2} }}$, and $K_{B}$ is Boltzmannn constant and $.d$ is particle diameter.

$$B\left( x \right) = B_{0} (x + y + c)^{{\frac{n - 1}{2}}} ,V_{w} = a(x + y + c)^{n}$$

15 $$U_{w} = a(x + y + c)^{{\frac{n - 1}{2}}} ,\;T_{w} = T_{\infty } + T_{0} (x + y + c)^{{\frac{1 - n}{2}}}$$

$$C_{w} = C_{\infty } + C_{0} (x + y + c)^{{\frac{1 - n}{2}}}$$

The transformation have been introduced^28^

$$u = a(x + y + c)^{n} f\left( \eta \right),\;\;v = a(x + y + c)^{n} g^{\prime \prime } \left( \eta \right),$$

16 $$w = - \sqrt {\frac{2av}{{n + 1}}} (x + y + c)^{{\frac{n - 1}{2}}} \left[ {\eta \left( {\frac{n - 1}{2}} \right)\left( {g^{\prime } + f^{\prime } } \right) + \frac{n + 1}{2}\left( {f + g} \right)} \right]$$

17 $$\eta = z\sqrt {\frac{{\left( {n + 1} \right)a}}{2v}} (x + y + c)^{{\frac{n - 1}{2}}} ,\;\;\theta = \frac{{T - T_{\infty } }}{{\left( {T_{w} \left( x \right) - T_{\infty } } \right)}}, \;\;\phi = \frac{{C - C_{\infty } }}{{\left( {C_{w} \left( x \right) - C_{\infty } } \right)}}$$

In view of Eqs. (16) and (17), Eqs. (8)–(11) corresponding to the boundary (13) become dimensionless as given below:

18 $$\begin{aligned} \left( {\frac{n + 1}{2}} \right)f^{\prime \prime } + \beta \left[ {\left\{ {2n\left( {n + 1} \right)g^{\prime } + \left( {n + 1} \right)\left( {3n - 1} \right)f^{\prime } } \right\}f^{\prime \prime \prime } - \frac{1}{2}\left\{ {\left( {g + f} \right)(n + 1)^{2} + \left( {n^{2} - 1} \right)\eta g^{\prime } } \right\}f^{{\left( {iv} \right)}} } \right. \hfill \\ \left. { - \frac{{\left( {n + 1} \right)\left( {3n - 1} \right)}}{2}\left( {f^{\prime \prime } - g^{\prime \prime } } \right)f^{\prime \prime } + \eta \frac{{\left( {n^{2} - 1} \right)}}{2}\left( {f^{\prime \prime } g^{\prime \prime \prime } + 2f^{\prime \prime \prime } g^{\prime \prime } } \right)} \right] - Mf^{\prime } - \left[ {nf^{\prime } g^{\prime } + nf^{^{\prime}2} - \frac{{\left( {n + 1} \right)}}{2}\left( {f + g} \right)f^{\prime \prime } } \right] = 0 \hfill \\ \end{aligned}$$

19 $$\begin{aligned} & \left( {\frac{n + 1}{2}} \right)g^{\prime \prime \prime } + \beta \left[ {\left\{ {2n\left( {n + 1} \right)f + \left( {3n - 1} \right)\left( {n + 1} \right)g^{\prime } } \right\}g^{\prime \prime \prime } - \frac{1}{2}\left\{ {\left( {f + g} \right)(n + 1)^{2} + \left( {n^{2} - 1} \right)\eta f^{\prime } } \right\}g^{{\left( {iv} \right)}} } \right. \\ & \quad \left. { - \frac{{\left( {3n - 1} \right)\left( {n + 1} \right)}}{2}\left( {g^{\prime \prime } - f^{\prime \prime } } \right)g^{\prime \prime } + \eta \frac{{\left( {n^{2} - 1} \right)}}{2}\left( {g^{\prime \prime } f^{\prime \prime \prime } + 2g^{\prime \prime \prime } f^{\prime \prime \prime } } \right)} \right] - Mg^{\prime } - \left[ {nf^{\prime } g^{\prime } + ng^{\prime 2} - \frac{{\left( {n + 1} \right)}}{2}\left( {f + g} \right)g^{\prime \prime } } \right] = 0 \\ \end{aligned}$$

20 $$\Pr \left( {\frac{1 - n}{{n + 1}}\left( {f^{\prime } + g^{\prime } } \right)\theta - \left( {f + g} \right)\theta^{\prime } + \frac{2}{n + 1}Q\theta + Nb\theta^{\prime } \phi^{\prime } + Nt\theta^{\prime 2} } \right) + \frac{2}{n + 1}\left( {L^{*} f^{\prime } + M^{*} \theta } \right) + \left( {1 + \left( \frac{4}{3} \right)R} \right)\theta^{\prime \prime } = 0$$

21 $$\phi^{\prime \prime } + Sc\left( {\left( {f + g} \right)\phi^{\prime } - \frac{{\left( {1 - n} \right)}}{n + 1}\left( {f + g^{\prime } } \right)\phi - \frac{2}{n + 1}\delta \phi } \right) + \frac{Nt}{{Nb}}\theta^{\prime \prime } = 0$$

where

22 $$\begin{aligned} {\text{Pr}} &= \frac{{\mu C_{p} }}{k},\;\;Q = \frac{{Q_{0} \left( {x + y + c} \right)}}{{\rho C_{p} U_{w} }},\;\;Sc = \frac{v}{{D_{B} }},\;R = \frac{{16\sigma^{*} T_{\infty }^{3} }}{{3kk^{*} }},\;\;M = \frac{{\sigma B_{0}^{2} }}{{a\rho_{f} }},\beta = \frac{{\alpha_{1} U_{w} }}{{2v\rho_{f} }}, \\ Nt = & \frac{{\tau D_{T} \left( {T_{w} - T_{\infty } } \right)}}{{T_{\infty } \left( {\mu C_{p} } \right)_{f} }},\delta = \frac{{K_{1} \left( {x + y + c} \right)}}{{U_{w} }},\;Nb = \frac{{\tau D_{B} \left( {C_{w} - C_{\infty } } \right)}}{{\left( {\mu C_{p} } \right)_{f} }}. \\ \end{aligned}$$

With conditions are

23 $$\begin{aligned} f\left( 0 \right) = & \lambda \left[ {1 + b_{1} f^{\prime \prime } \left( 0 \right)} \right]\left( {\frac{1 - n}{{n + 1}}} \right),\;f^{\prime } \left( 0 \right) = \lambda_{1} + b_{1} f^{\prime \prime } \left( 0 \right) \\ g\left( 0 \right) = & \lambda \left[ {1 + b_{1} g^{\prime \prime } \left( 0 \right)} \right]\left( {\frac{1 - n}{{n + 1}}} \right),\;g^{\prime } \left( 0 \right) = 1 + b_{1} g^{\prime \prime } (0) \\ \theta \left( 0 \right) = & \left[ {1 + b_{2} \theta^{\prime } \left( 0 \right)} \right],\;\phi \left( 0 \right) = \left[ {1 + b_{3} \phi^{\prime } \left( 0 \right)} \right] \\ f^{\prime}\left( \infty \right) = & 0,f^{\prime } \left( \infty \right) = 0,\;g^{\prime } \left( \infty \right) = 0 \\ g^{\prime\prime}\left( \infty \right) = & 0,\theta \left( \infty \right) = 0,\;\phi \left( \infty \right) = 0 \\ \end{aligned}$$

With

24 $$b_{1} = \zeta_{1} \left( {\frac{{2 - f_{1} }}{{f_{1} }}} \right)\sqrt {\frac{{\left( {n + 1} \right)a}}{2v}} ,\;\;b_{2} = \zeta_{2} \left( {\frac{2 - b}{b}} \right)\sqrt {\frac{{\left( {n + 1} \right)a}}{2v}} ,{ }b_{3} = \zeta_{3} \left( {\frac{2 - m}{m}} \right)\sqrt {\frac{{\left( {n + 1} \right)a}}{2v}} ,\lambda = A\sqrt {\frac{{\left( {n + 1} \right)a}}{2v}} .$$

The physical parameters of concern are.

**Skin friction (SF)**

25 $$\begin{aligned} {\text{Re}}_{x}^{\frac{1}{2}} C_{Fx} &= 2\left( {\frac{n + 1}{2}} \right)^{\frac{1}{2}} \left( {f^{\prime \prime } \left( 0 \right) + 2\beta \left( {2nf^{\prime \prime } \left( 0 \right)\left( {f^{\prime } \left( 0 \right) + g^{\prime } \left( 0 \right)} \right)} \right.} \right., \hfill \\ & \quad+ n\left( {f^{\prime } \left( 0 \right)f^{\prime \prime } \left( 0 \right) + g^{\prime } \left( 0 \right)g^{\prime \prime } \left( 0 \right)} \right)\left. {\left. { - \left( {\frac{n + 1}{2}} \right)\left( {f\left( 0 \right) + g\left( 0 \right)} \right)f^{\prime \prime } \left( 0 \right)} \right)} \right), \hfill \\ \end{aligned}$$

26 $$\begin{aligned} {\text{Re}}_{x}^{\frac{1}{2}} C_{Fy} &= 2\left( {\frac{n + 1}{2}} \right)^{\frac{1}{2}} \left( {2\beta \left( {2ng^{\prime \prime } \left( 0 \right)\left( {f\left( 0 \right) + g^{\prime } \left( 0 \right)} \right)} \right.} \right. \hfill \\ & \quad \left. {\left. { + n\left( {f^{\prime } \left( 0 \right)f^{\prime \prime } \left( 0 \right) + g^{\prime } \left( 0 \right)g^{\prime \prime } \left( 0 \right)} \right) - \left( {\frac{n + 1}{2}} \right)\left( {f\left( 0 \right) + g\left( 0 \right)} \right)g^{\prime \prime \prime } \left( 0 \right)} \right)} \right) \hfill \\ \end{aligned}$$

**Nusselt number (NN)**

27 $$Re_{x}^{{ - \frac{1}{2}}} Nu_{x} = - \left( {1 + R} \right)\left( {\frac{n + 1}{2}} \right)^{\frac{1}{2}} \theta^{\prime}\left( 0 \right),$$

**Sherwood number (SN)**

28 $$Re_{x}^{{ - \frac{1}{2}}} Sh_{x} = - \left( {\frac{n + 1}{2}} \right)^{\frac{1}{2}} \phi^{\prime } \left( 0 \right).$$

where $R{e}_{x}=\frac{{U}_{w}(x)(x+y+c)}{{v}_{f}}$ is the Reynolds number.

## Numerical solutions

The modeled highly nonlinear differential Eqs. (18)–(21) corresponding to the boundary constraints (23) are elucidated numerically by the bvp4c algorithm in Matlab software which is executed in three-phase collocation procedure as discussed by Rehman et al.^19^. Through collocation polynomial, a uniform 4th-order solution is executed like solution C1-continuous in the integration interval. In the second phase a collocation approach implements a mesh to splitting the interval into sub-intervals. The solver assurances a solution of the modeling system. The on each subinterval, the error is estimated through a solver. The process is reiterated with mesh modification if the needed tolerance is not encountered. The flow chart of the bvp4c technique is expressed by Fig. 1.

Frist, we want to change the Eqs. (18)–(21) corresponding to the boundary condtions (23) to first order differential equations by using the following phases.

Phase-I: New transformations are planned for the given modeled nonlinear ODEs given by Eqs. (18)–(21):

29 $$\begin{aligned} f = & z_{1} , \;f^{\prime } = z_{2} , \;f^{\prime \prime } = z_{3} ,\;f^{\prime \prime \prime } = z_{4} g = z_{5} , \;g^{\prime } = z_{6} , \;g^{\prime \prime } = z_{7} , \\ g^{\prime \prime \prime } = & z_{8} ,\;\theta = z_{9} ,\theta^{\prime } = z_{10} , \phi = z_{11} , \;and\; \phi^{\prime} = z_{12} . \\ \end{aligned}$$

Phase-II: Reduce the Eqs. (18)–(21) with Eqs. (23) into first order by using the Eq. (29).

30 $$\begin{gathered} \left( {\frac{n + 1}{2}} \right)z_{3} + \beta \left[ {\left\{ {2n\left( {n + 1} \right)z_{5} + \left( {n + 1} \right)\left( {3n - 1} \right)z_{2} } \right\}z_{4} - \frac{1}{2}\left\{ {\left( {z_{4} + z_{1} } \right)(n + 1)^{2} + \left( {n^{2} - 1} \right)\eta g^{\prime } } \right\}z_{4}^{\prime } } \right. \hfill \\ \left. { - \frac{{\left( {n + 1} \right)\left( {3n - 1} \right)}}{2}\left( {z_{3} - z_{6} } \right)z_{3} + \eta \frac{{\left( {n^{2} - 1} \right)}}{2}\left( {z_{3} z_{6} + 2z_{5} z_{6} } \right)} \right] - Mz_{2} , - \left[ {nz_{2} z_{5} + nz_{2}^{2} - \frac{{\left( {n + 1} \right)}}{2}\left( {z_{1} + z_{5} } \right)z_{3} } \right] = 0 \hfill \\ \end{gathered}$$

31 $$\begin{gathered} \left( {\frac{n + 1}{2}} \right)z_{7} + \beta \left[ {\left\{ {2n\left( {n + 1} \right)z_{1} + \left( {3n - 1} \right)\left( {n + 1} \right)z_{6} } \right\}z_{8} - \frac{1}{2}\left\{ {\left( {z_{1} + z_{5} } \right)(n + 1)^{2} + \left( {n^{2} - 1} \right)\eta z_{2} } \right\}z_{8}^{\prime } } \right. \hfill \\ \left. { - \frac{{\left( {3n - 1} \right)\left( {n + 1} \right)}}{2}\left( {z_{7} - z_{3} } \right)z_{7} + \eta \frac{{\left( {n^{2} - 1} \right)}}{2}\left( {z_{7} z_{4} + 2z_{8} z_{4} } \right)} \right] - Mz_{6} - \left[ {nz_{2} z_{6} + nz_{6}^{2} - \frac{{\left( {n + 1} \right)}}{2}\left( {z_{1} + z_{5} } \right)z_{7} } \right] = 0 \hfill \\ \end{gathered}$$

32 $${\text{Pr}}\left( {\frac{1 - n}{{n + 1}}\left( {z_{2} + z_{6} } \right)z_{9} - \left( {z_{1} + z_{5} } \right)z_{10} + \frac{2}{n + 1}Qz_{9} + Nbz_{10} z_{12} + Ntz_{10}^{2} } \right) + \frac{2}{n + 1}\left( {L^{*} z_{2} + M^{*} z_{9} } \right) + \left( {1 + \left( \frac{4}{3} \right)R} \right)z_{10}^{\prime} = 0$$

32 $$z_{12}^{\prime} + Sc\left( {\left( {z_{1} + z_{5} } \right)z_{12} - \frac{{\left( {1 - n} \right)}}{n + 1}\left( {z_{1} + z_{6} } \right)z_{11} - \frac{2}{n + 1}\delta z_{11} } \right) + \frac{Nt}{{Nb}}z_{10}^{\prime} = 0$$

Phase-III: Transformed the boundary constraints in Eq. (23) in term of new transformations which are given in Eq. (29)

33 $$\begin{gathered} \left( {z_{1} } \right)_{a*} = \lambda \left[ {1 + b_{1} \left( {z_{3} } \right)_{a*} } \right]\left( {\frac{1 - n}{{n + 1}}} \right),\left( {z_{2} } \right)_{a*} = 1 + b_{1} \left( {z_{3} } \right)_{a*} \hfill \\ \left( {z_{5} } \right)_{a*} = \lambda \left[ {1 + b_{1} \left( {z_{7} } \right)_{a*} } \right]\left( {\frac{1 - n}{{n + 1}}} \right),\left( {z_{6} } \right)_{a*} = 1 + b_{1} \left( {z_{7} } \right)_{a*} \hfill \\ \left( {z_{9} } \right)_{a*} = \left[ {1 + b_{2} \left( {z_{10} } \right)_{a*} } \right],\left( {z_{11} } \right)_{a*} = \left[ {1 + b_{3} \left( {z_{12} } \right)_{a*} } \right] \hfill \\ \left( {z_{2} } \right)_{b*} = 0,\left( {z_{2} } \right)_{b*} = 0,\left( {z_{6} } \right)_{b*} = 0 \hfill \\ \left( {z_{7} } \right)_{b*} = 0,\left( {z_{9} } \right)_{b*} = 0,\left( {z_{12} } \right)_{b*} = 0 \hfill \\ \end{gathered}$$

**Figure 2:**
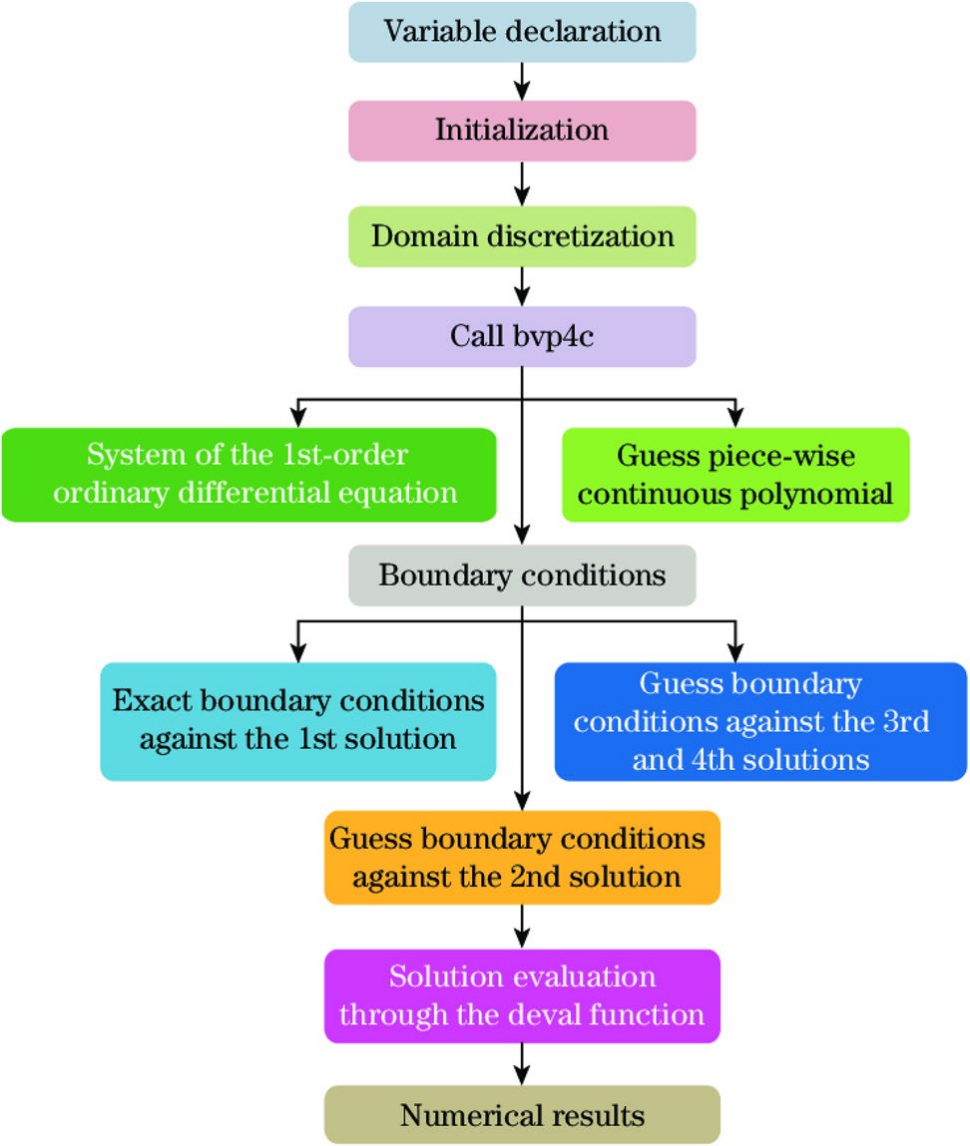
Flow chart of bvp4c.

In above Eq. (33), the location of the surface at $\eta =0$ is denoted by subscript $a*$ while the subscript $b*$ signifies the distance from the surface for a specific value of $\eta .$ In the present study we set the range as $0\le \eta \le 7.$ For two different initial estimates we get dual solution by using bvp4c algorithm. The first solution is obtained through initial prediction which is quite open-ended. But this is not always possible to get the second solution. This processes is repeated again and again until we get the solution which satisfy the boundary conditions at infinity i.e., Eq. (23) asymptotically converge. The flow chart of the numerical technique is represented in Fig. 2.

## Results and discussion

The basic flow equations are modeled in mathematical formulations for the velocities, temperature, and concentration profiles in terms of PDEs. The PDEs are altered to non-dimensional ODEs through the similarity conversions. The computational results have been obtained through the bvp4c algorithm in Matlab software. The procedure for the bvp4c is defined in Sect. 3.

Further, to confirm the precision of the present algorithm, the present finding for the local Nusselt number (LNN) $-\mathrm{\theta^ {\prime}}(0)$ is validated with the reported work of Gayatri et al.^28^. This comparative study is given in Table 1 for the assigned values of Prandtl number. This table shows a good settlement with the current homework, therefore, validating the consistency of the existing method.

**Table 1:** Validation of the present study by comparing with published work reported by Gayatri et al.^28^ for various values of $\mathrm{Pr}$ and n = 0.5 and taking the rest of physical factors to zero.

Pr	Local Nusselt number	
	Gayatri et al.^28^	Current work
0.5	0.3264300	0.3264201
1.0	0.5250730	0.5250521
1.5	0.6917360	0.6917250
2.0	0.8350920	0.83507103

**Figure 3:**
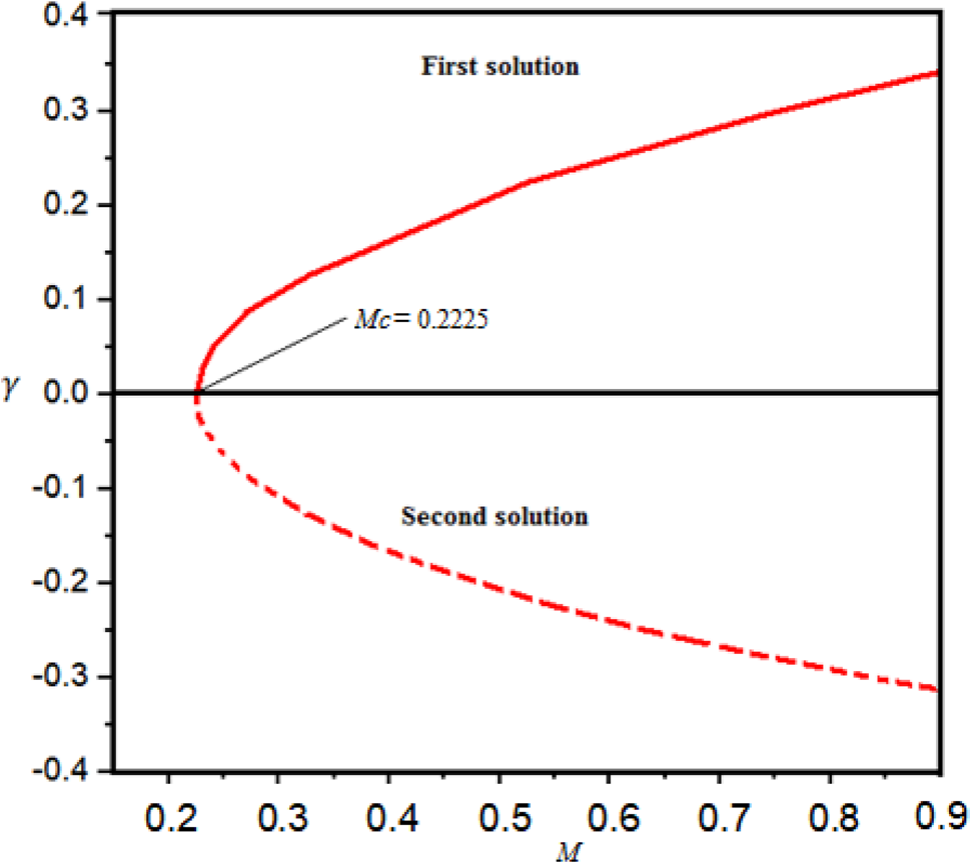
Smallest eigenvalues $\gamma$ with magnetic parameter $M.$

The Mathematical model given in Eqs. (18)–(21) with (23) offered non-unique solutions for some emerging parameters. As a result, a stability study is achieved to conclude which solution is most reliable. Therefore, the fundamental equations given in Eqs. (7)–(11) with Eq. (13) are made unsteady. The stability solution is obtained of the steady flow $f\left(\eta \right)={f}_{0}\left(\eta \right), g\left(\eta \right)={g}_{0}\left(\eta \right), \theta \left(\eta \right)={\theta }_{0}\left(\eta \right), and\,\phi \left(\eta \right)={\phi }_{0}\left(\eta \right)$ by expressing $f\left(\eta , \tau \right)={f}_{0}\left(\eta \right)+{e}^{-\gamma \tau }J\left(\eta ,\tau \right)$,$g\left(\eta , \tau \right)={g}_{0}\left(\eta \right)+{e}^{-\gamma \tau }F\left(\eta ,\tau \right)$,$\theta \left(\eta , \tau \right)={\theta }_{0}\left(\eta \right)+{e}^{-\gamma \tau }G\left(\eta ,\tau \right)$, $\phi \left(\eta \right)={\phi }_{0}\left(\eta \right)+{e}^{-\gamma \tau }H\left(\eta ,\tau \right)$ which satisfy the boundary flow problem as proposed by Merkin^31^ and Wiedman et al.^32^. Here, $\gamma$ is an undetermined eigenvalues and $J\left(\eta ,\tau \right)$,$F\left(\eta ,\tau \right)$, $G\left(\eta ,\tau \right)$ and $H\left(\eta ,\tau \right)$ are relatively small to ${f}_{0}\left(\eta \right),$ ${\theta }_{0}\left(\eta \right),$ ${g}_{0}\left(\eta \right)$, and ${\phi }_{0}\left(\eta \right)$. After simplification the resultant equations are solved numerically by bvp4c MATLAB's software. Figure 3 demonstrates the phenomena of the dual solutions, i.e., the stable (first solution) and unstable (second solution) solutions. Figure 3 shows the lowest eigenvalues $\gamma$ versus magnetic parameter $M$. We suggest that the first branch is reliable while the second branch is unstable based on the prior research. It is noteworthy that this approach is crucial for finding a stable solution when non-unique solutions exist, allowing for reliable flow behavior prediction.

**Figure 4:**
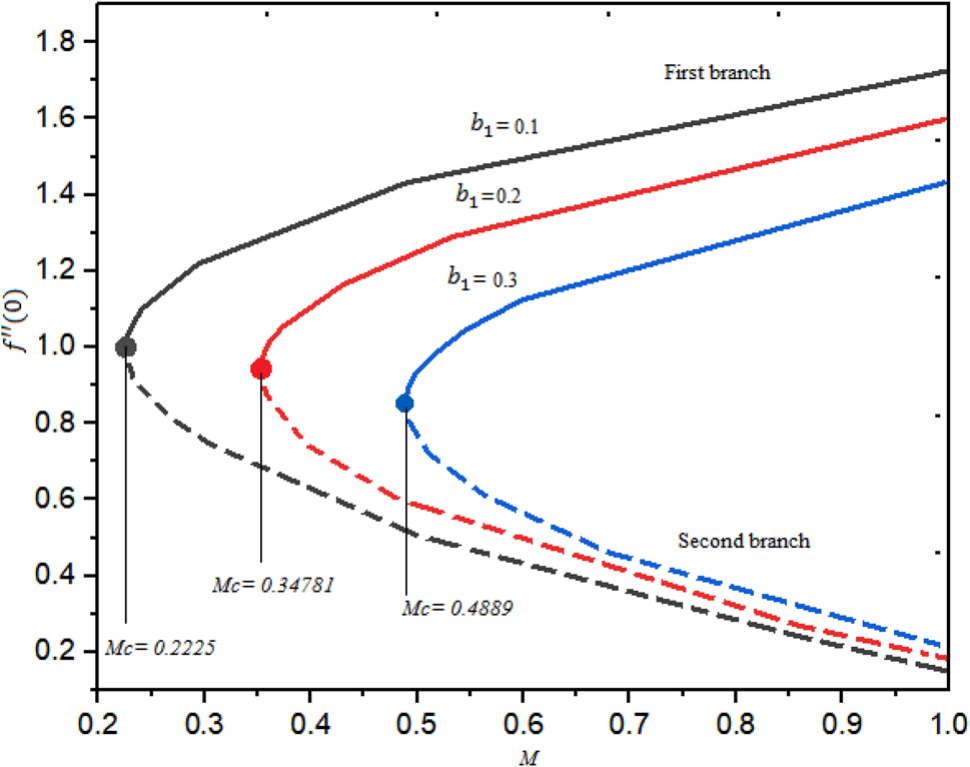
Reduced skin friction for numerous quantities of ${b}_{1}$ regarding $M.$

**Figure 5:**
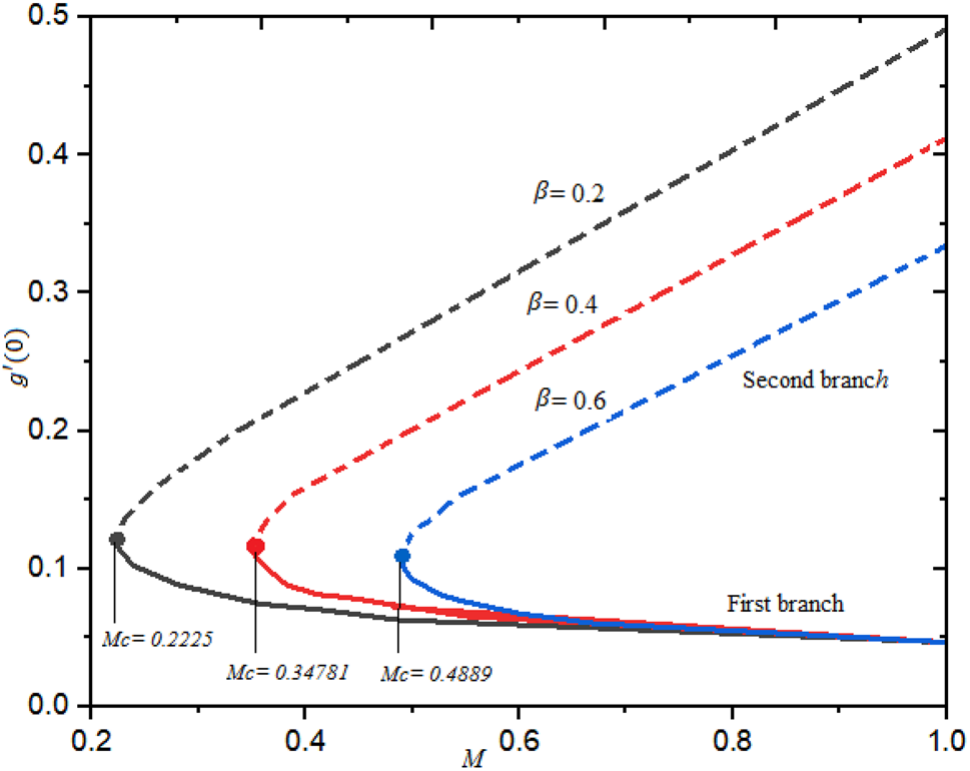
Local skin friction for numerous quantities of $\beta$ regarding $M.$

The dual branches occur when $M\ge {M}_{cj}$; j = 1, 2, 3, while no solution exists when $M<{M}_{cj}$. It is witnessed that for the increasing magnitude of the slip factor ${b}_{1}$, the profile of $f^{\prime\prime}(0)$ declines with the increasing magnitude of $M$ in the second branch as shown in Fig. 4. In addition, an opposite trend is detected in the second branch with the growth of ${b}_{1}$ regarding $M$. Similalry, the influence of the second-grade factor $\beta$ on the $g^{\prime}(0)$ is demonstrated in Fig. 5 for a different order of $M$. In this case also dual branches of solution are scrutinized. It is witnessed that the curve of $g^{\prime}(0)$ is decelerated as the factor $\beta$ is augmented.

**Figure 6:**
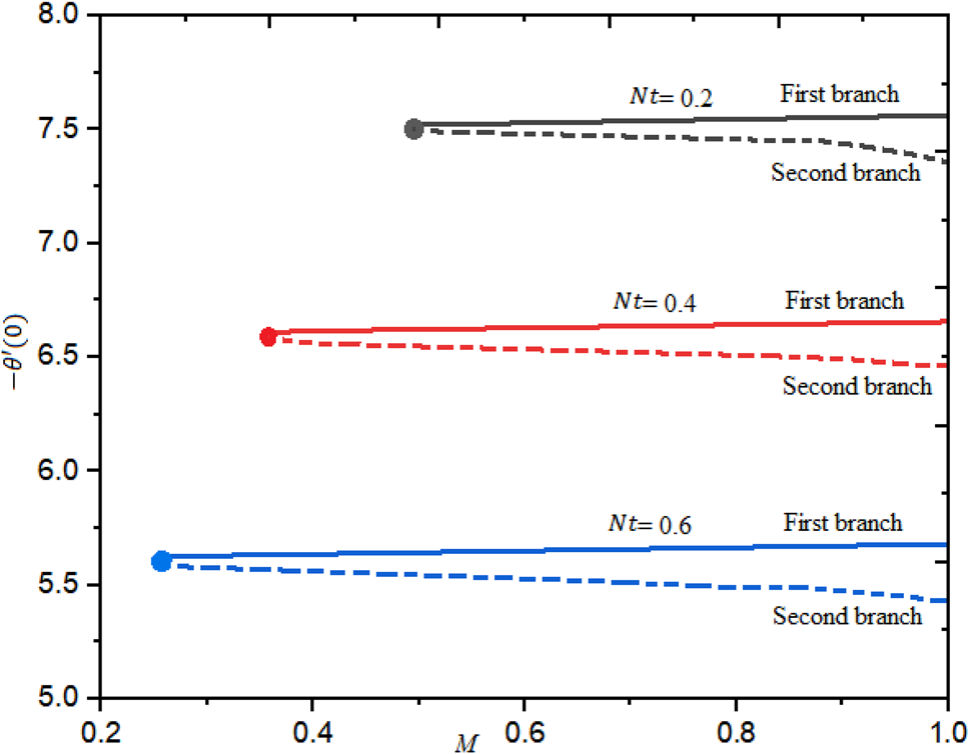
Heat rate for numerous quantities of $\mathrm{Nt}$ regarding $M.$

Figure 6 describes the fluctuation of heat transmission rate for numerous magnitudes of thermophoresis factor $Nt$ using the different order of approximation of magnetic field. Here, in this situation dual branches of solution are established. The upper branch is higher as related to the second branch. The rate of heat transfer is enhanced as the thermophoresis factor is decreased.

**Figure 7:**
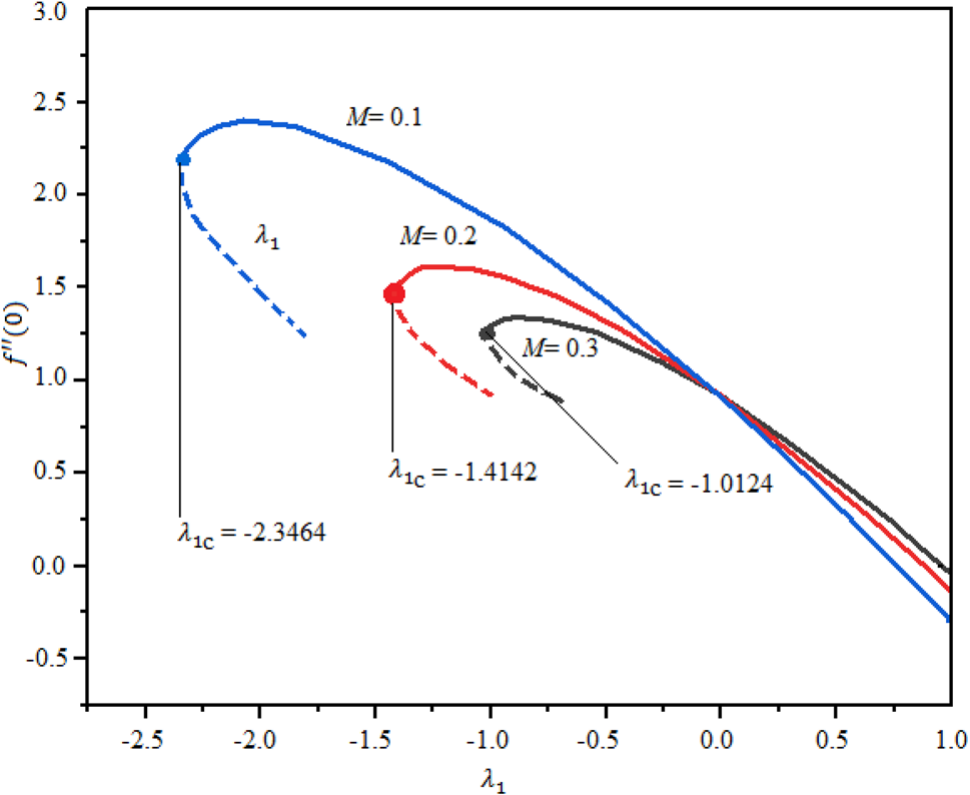
Local skin friction with suction $({\lambda }_{1}<0)$ and injection $(v1>0)$ for $\phi .$

**Figure 8:**
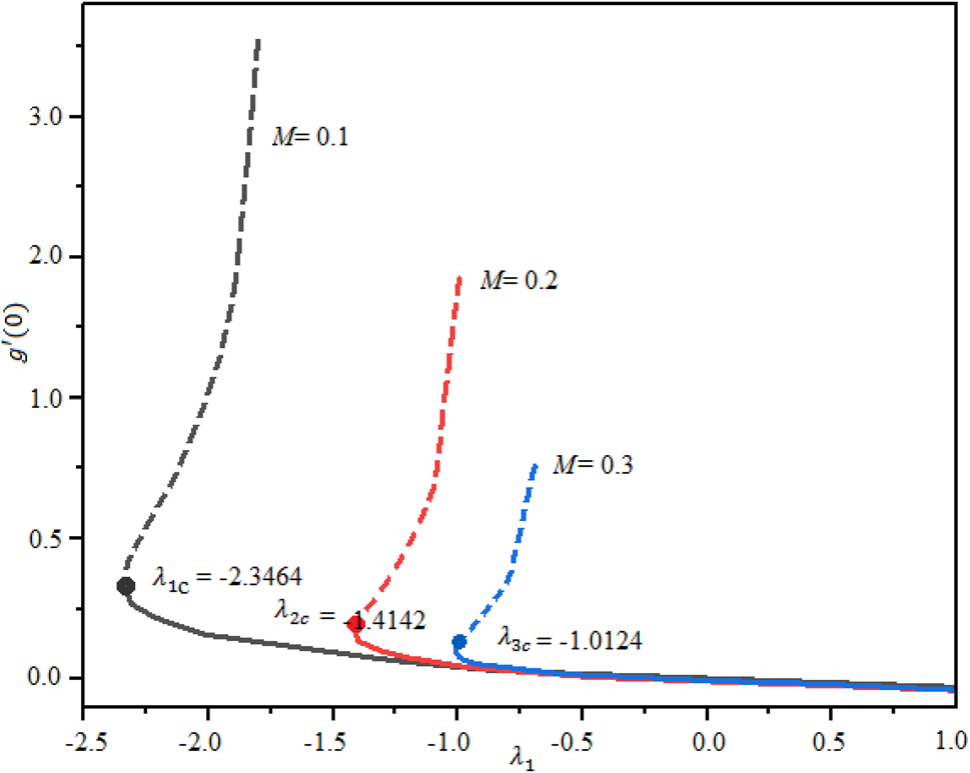
Nusselt number with suction $(v1<0)$ and injection $(v1>0)$ for $\phi .$

**Figure 9:**
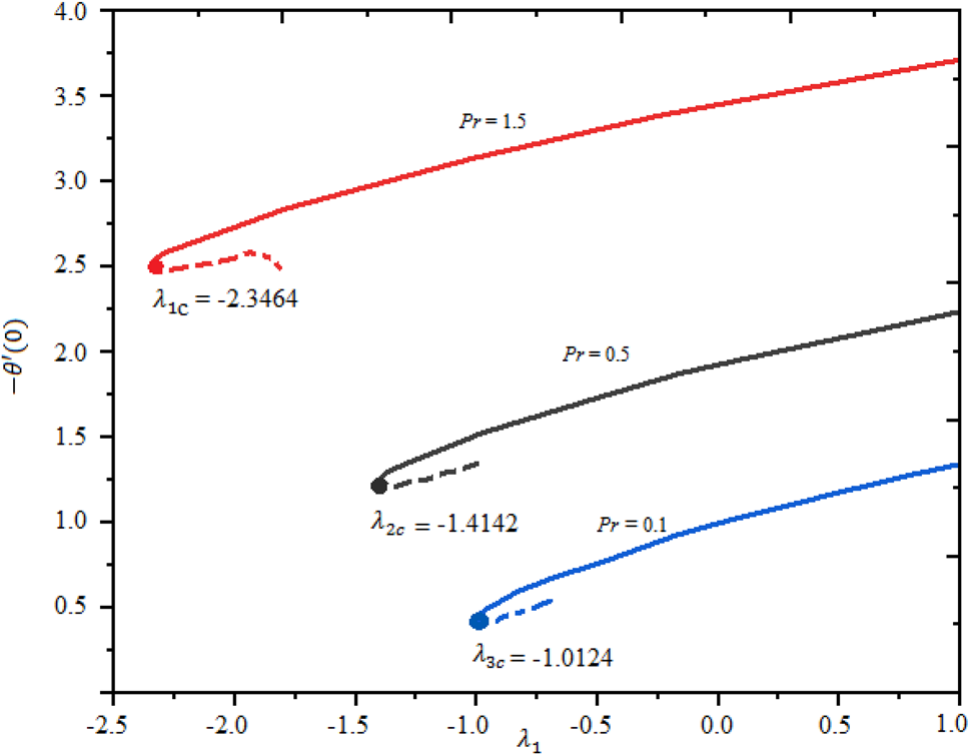
Nusselt number with *B1*versis suction $(v1<0)$ and injection $\left(v1>0\right).$

The impact of magnetic factor $M$ and $\mathrm{Pr}$ on the LSF and heat transferring is revealed in Figs. 7, 8 and 9 against extending/contracting parameter ${\lambda }_{1}$. It is detected that reduced skin friction reduces as the fluid flow travels towards the stretching sheet in both solutions. Dual behavior is observed for the profile $f^{\prime\prime}(0)$. In the domain ${-2.5\le \lambda }_{1}\le 0$, the profile $f^{\prime\prime}(0)$ decreases while in the range ${0\le \lambda }_{1}\le 1.0$; increasing behavior is observed for the profile $f^{\prime\prime}(0)$. Simply say that the LSF is declining function of $M$ in the stretching case while growing function for the case of shrinking as shown in Fig. 7. The critical values for $M=$ 0.1, 0.3, and 0.3 are ${\lambda }_{c1}=$− 2.3464, − 1.4142, and − 1.0124, individually and no solution exist at ${\lambda }_{1}\le {\lambda }_{cj}$; j = 1, 2,3. Similarly, the profile $g^{\prime}(0)$ decreases as $M$ enhances. The LSF is advanced for the first branch as related to the second branch. This variation is more significant in the domain ${-2.5\le \lambda }_{1}\le -0.1$ and negligible in ${0\le \lambda }_{1}\le 1$ as portrayed in Fig. 8. Figure 9 demonstrated that the heat rate is increased as the quantity of Prandtl number increases. The transferrant of heat is higher for the extending surface as related to the contracting surface for both branches.

In above Figs. 3,4, 5, 6, 7, 8 and 9, stability of the solutions has been elaborated in detail. We observe that the first branch is reliable while the lower branch is unstable. In the following analysis we are interested only on the stable solutions. For this purpose Figs. 10, 11, 12, 13, 14, 15, 16, 17, 18, 19, 20, 21, 22, 23, 24, 25, 26 and 27 are made.

**Figure 10:**
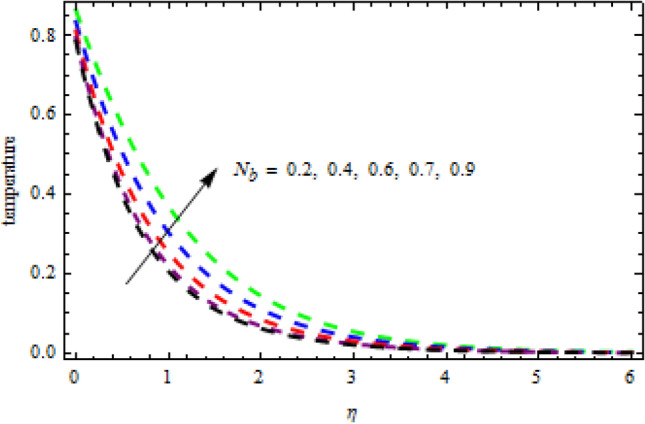
Impression of ${N}_{b}$ on $\theta \left(\eta \right).$

**Figure 11:**
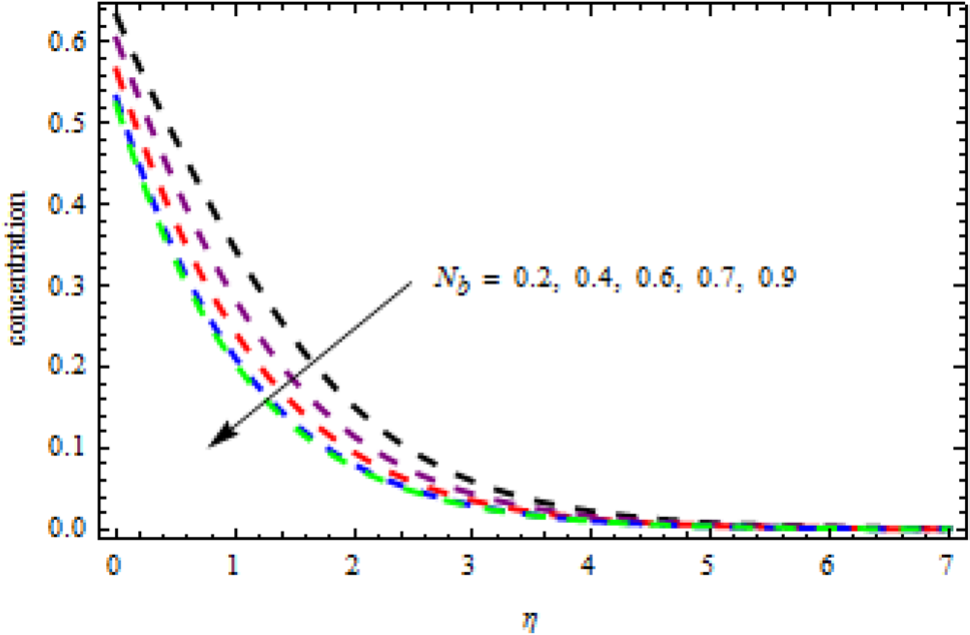
Consequence of ${N}_{b}$ on $\phi \left(\eta \right).$

**Figure 12:**
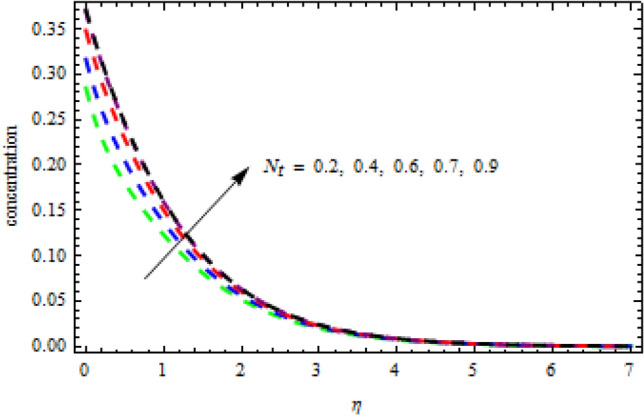
Impression of ${N}_{t}$ on $\phi \left(\eta \right).$

**Figure 13:**
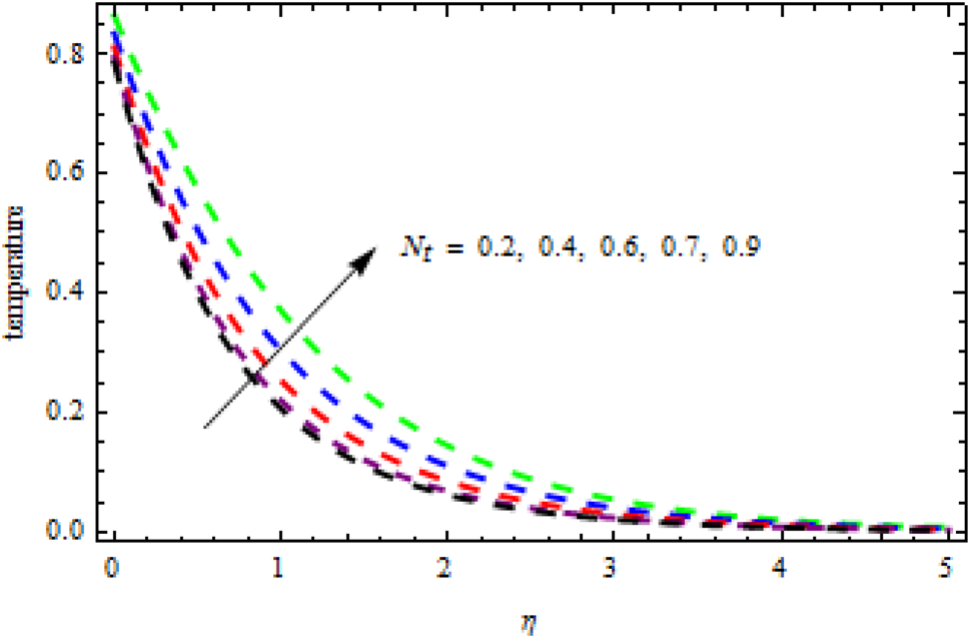
Consequence of ${N}_{t}$ on $\theta \left(\eta \right).$

**Figure 14:**
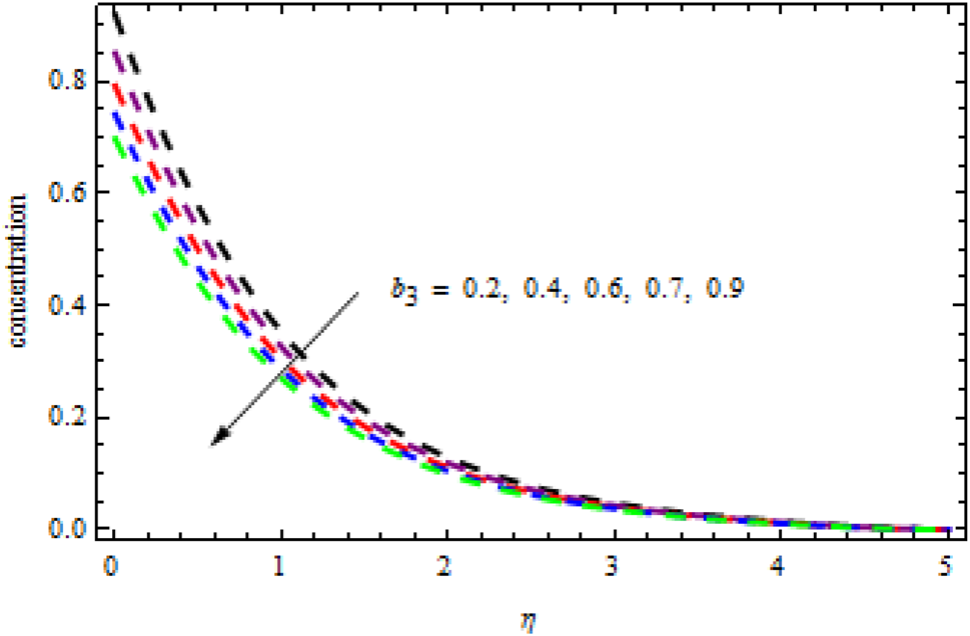
Impression of ${b}_{3}$ on $\phi \left(\eta \right).$

**Figure 15:**
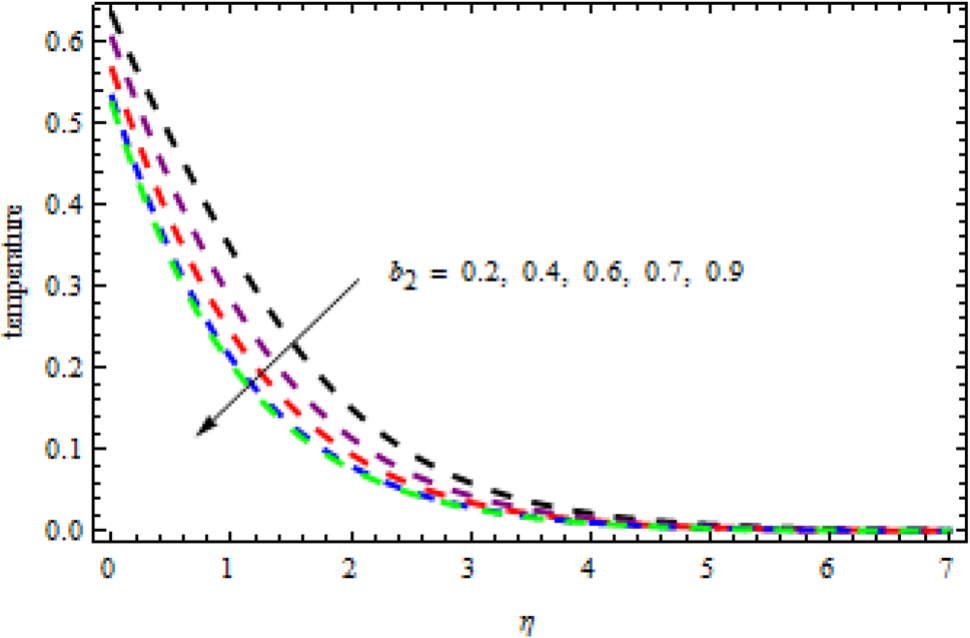
Consequenc of ${b}_{2}$ on $\theta \left(\eta \right).$

**Figure 16:**
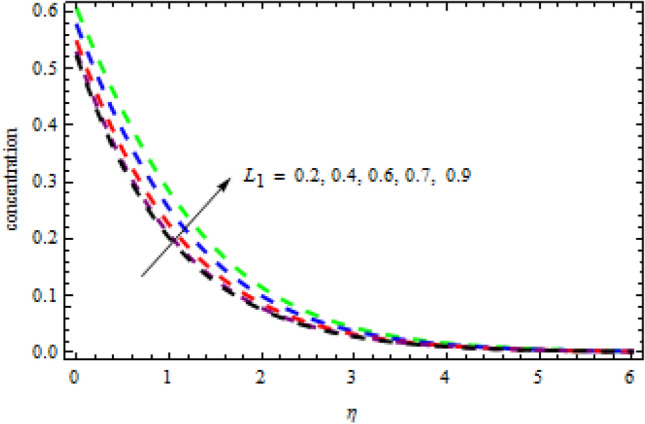
Impression of ${L}_{1}$ on $\phi \left(\eta \right).$

**Figure 17:**
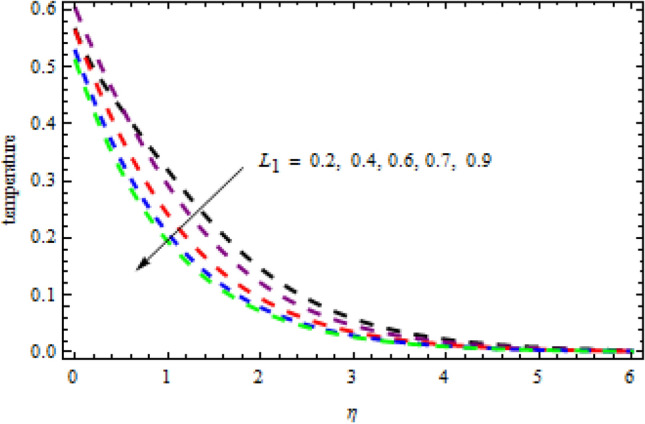
Impression of ${L}_{1}$ on $\theta \left(\eta \right).$

**Figure 18:**
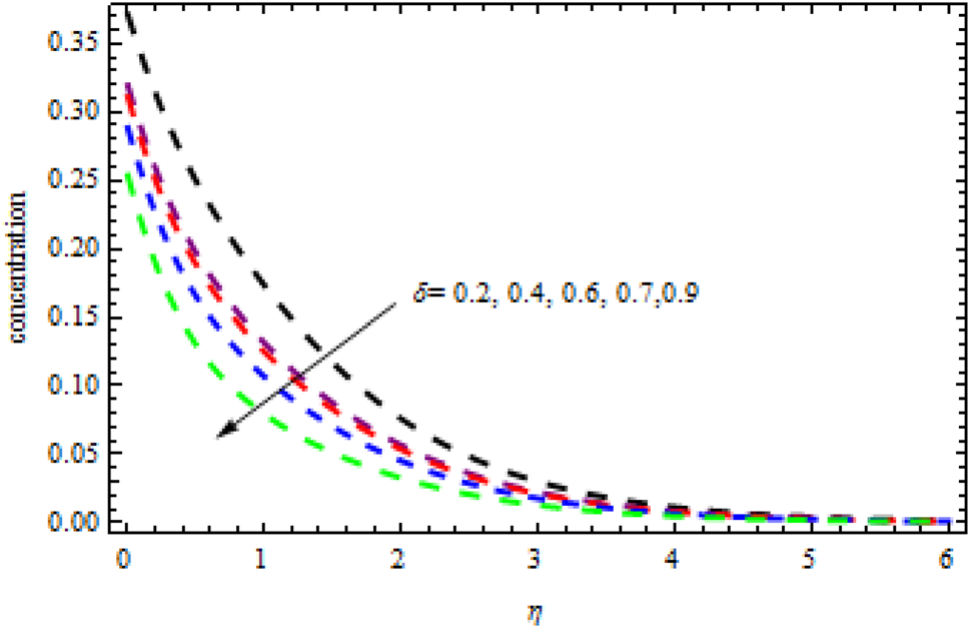
Impression of $\delta$ on $\phi \left(\eta \right).$

**Figure 19:**
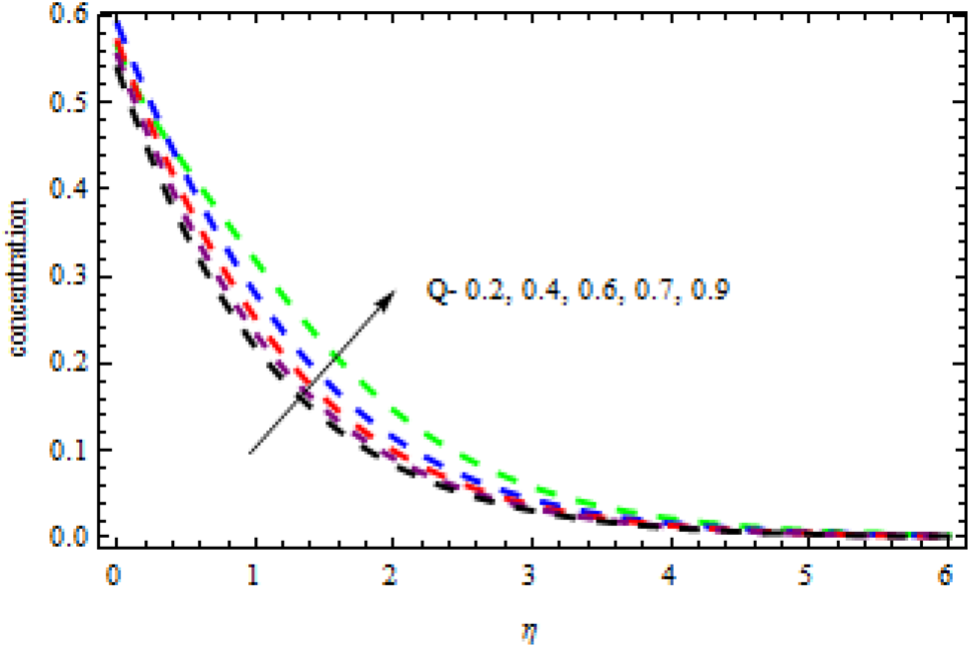
Impression of $Q$ on $\phi \left(\eta \right).$

**Figure 20:**
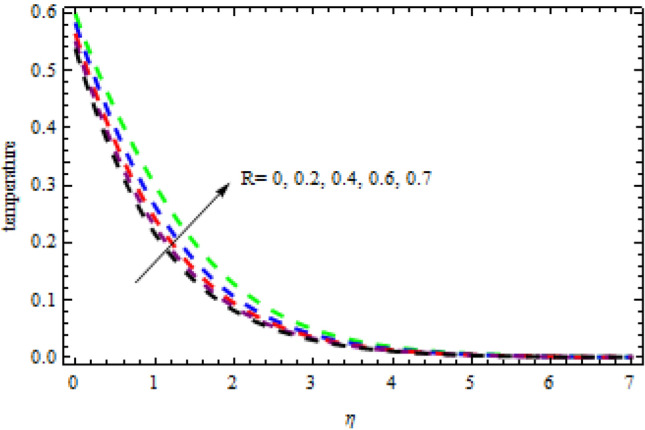
Impression of $R$ on $\theta \left(\eta \right).$

**Figure 21:**
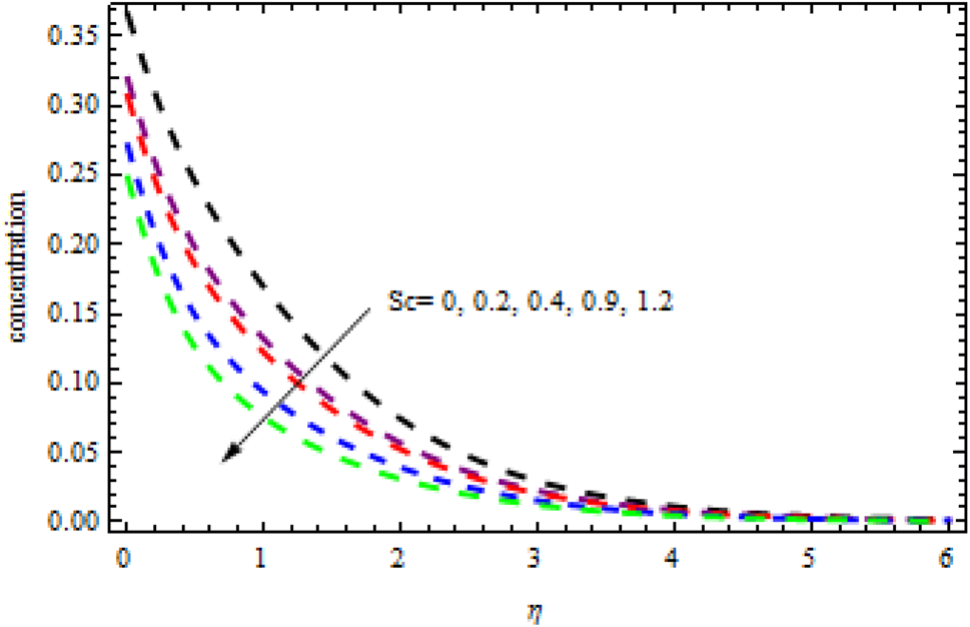
Impression of $Sc$ on $\phi \left(\eta \right).$

**Figure 22:**
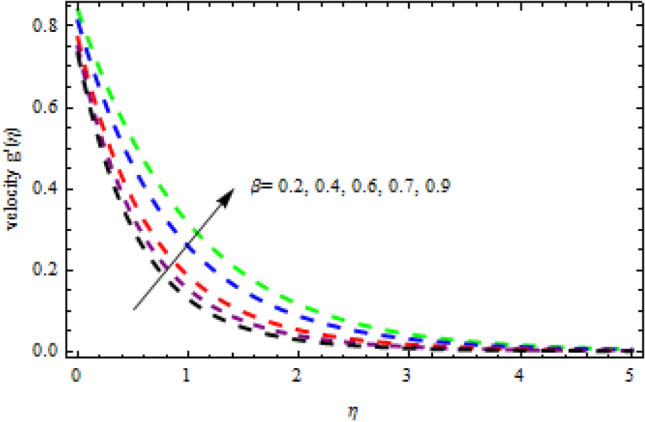
Impression of $\beta$ on $g^{\prime}\left(\eta \right).$

**Figure 23:**
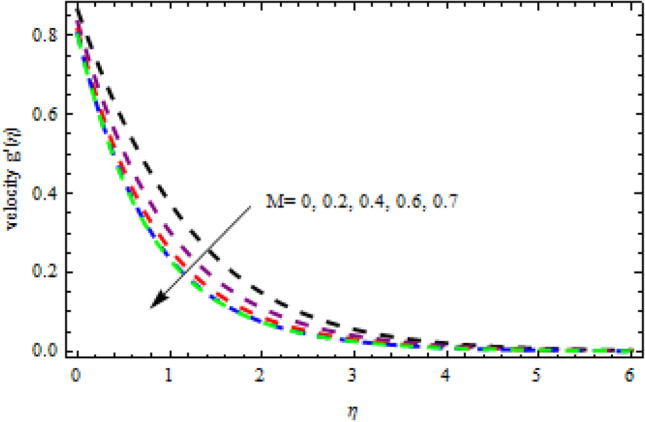
Impression of $M$ on $g^{\prime}\left(\eta \right).$

**Figure 24:**
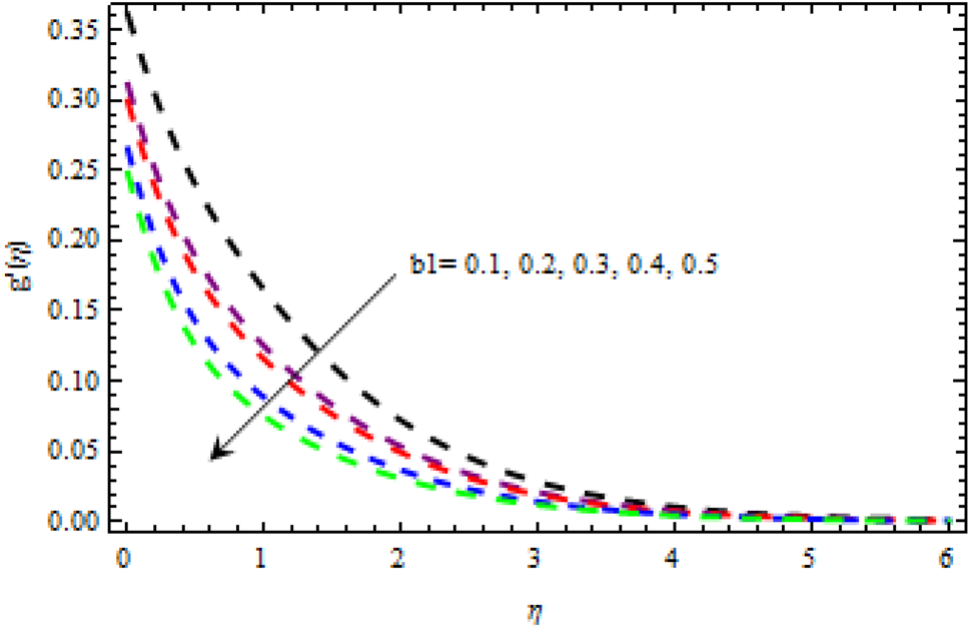
Impression of ${b}_{1}$ on $g^{\prime}\left(\eta \right).$

**Figure 25:**
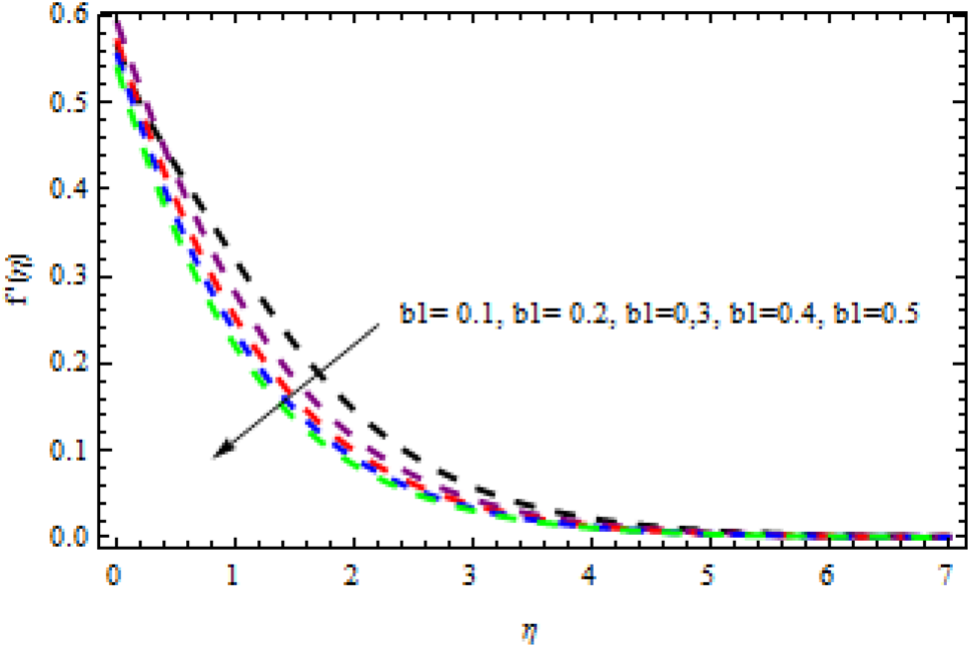
Impression of ${b}_{1}$ on $f^{\prime}\left(\eta \right).$

**Figure 26:**
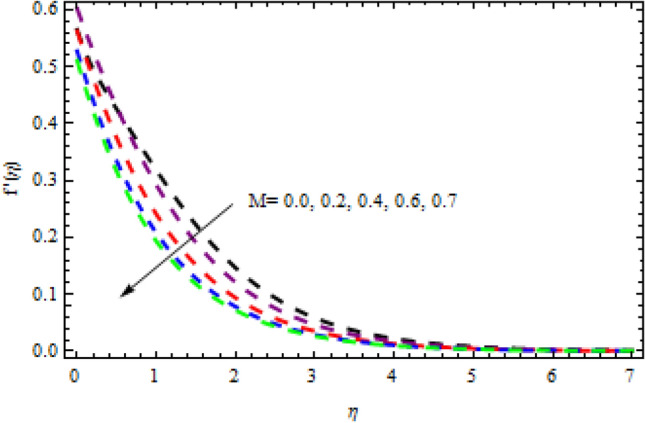
Impression of $M$ on $f^{\prime}\left(\eta \right).$

**Figure 27:**
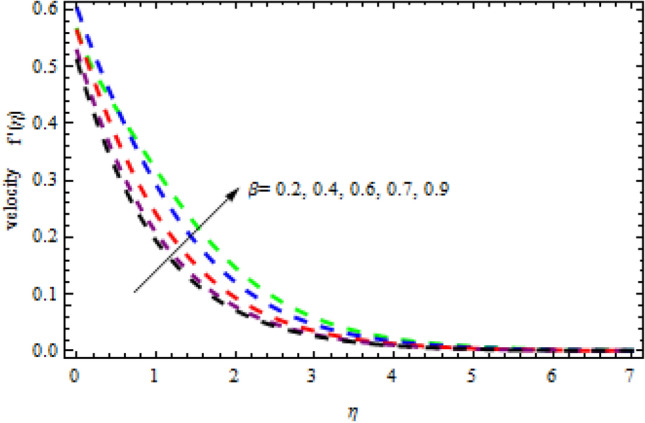
Impression of $\beta$ on $f{^\prime}\left(\eta \right).$

The influence of the important parameters like radiation $R,$ magnetic fields $M,$ Brownian motion $Nb$, chemical reactions $\delta$, thermophoresis $Nt,$ second-grade fluid factor $\beta$, Schmidt number $Sc$, velocity slip ${b}_{1}$, temperature jump ${b}_{2}$, heat sink/source ${M}^{*}$, and ${L}^{*}$, and concentration jump ${b}_{3}$ are all investigated. With the initial values of $Pr=2,Nt={M}^{*}=Q=$ $0.2,Nb=0.5,\delta =\beta ={b}_{2}=0.3,M=0.5,{b}_{1}=0.1,\lambda =0.3,{b}_{3}=$ $L=0.6,n=0.2,R=0.3,Sc=1.2$, the desire results are obtained. The consequence of Brownian motion $Nb$ on concentration and temperature is presented in Figs. 10 and 11, respectively. As the level of Brownian motion $Nb$ is raised, the temperature curves improve but the concentration decreases. Physically, when the temperature of a system increases, the rate at which the particles move due to Brownian motion also increases. Diffusion is the migration of particles in response to an applied concentration gradient. Higher-to-lower mass transmission rates have occurred. Both concentration and temperature are shown to be affected by the thermophoresis factor, as seen in Figs. 12 and 13, individually. The enhanced thermophoresis resulted in higher liquid temperatures and faster particle motion. Figures 14 and 15 demonstrated the influence of ${b}_{2}$ and ${b}_{3}$ factors on the concentration and temperature, correspondingly. It is revealed that the profiles of $\theta (\eta )$ and the $\phi (\eta )$ decelerated as the quantities of ${b}_{2}$ and ${b}_{3}$ are increased. Physically, owing to increasing the thermal jump, the temperature of the inner fluid is reduced which declines $\theta \left(\eta \right).$ Similarly, as ${b}_{3}$ is increased; the liquid mass transfer reduces which in results decline the $\phi (\eta )$ profile.

Figures 16 and 17 exhibition the impression of $L$ on profiles $\theta (\eta )$ and $\phi (\eta )$, respectively. It is detected that the concentration improves for the increasing quantities of $L$ while opposite influence is portrayed on temperature. The impact of chemical reactor $\delta$ on the $\phi (\eta )$ field is publicized in Fig. 18. It is revealed that due to increasing magnitude of $\delta$, the concentration curve improves in the channel. Physically, due to the presence of solute in the model at higher concentration, the collision of the particles improves in a given time interval which increases the reaction. The impression of heat generation on temperature is presented in Fig. 19. Higher levels of heat production led to more favorable temperature curves. As the temperature boosts up, the particles' kinetic energy boosts up because of random collisions of fluid particles, therefore, the heat generation improves. Figure 20 presents the influence of $R$ on the temperature curves. The temperature curves improve when the quantities of the radiation are improved. Physically, due to radiation drops on the fluid surface, the collision of the hot and cool particles are improved which enhances the inner temperature of the fluid.

Figure 21 reports the variation in Schmidt number on $\phi (\eta )$. $\phi (\eta )$ Shows a decline trend as the Schmidt number is increased. Physically; $\phi (\eta )$ the profile presents an inverse connection between the Schmidt number and Brownian diffusivity. Due to this the concentration declines.

**Figure 28:**
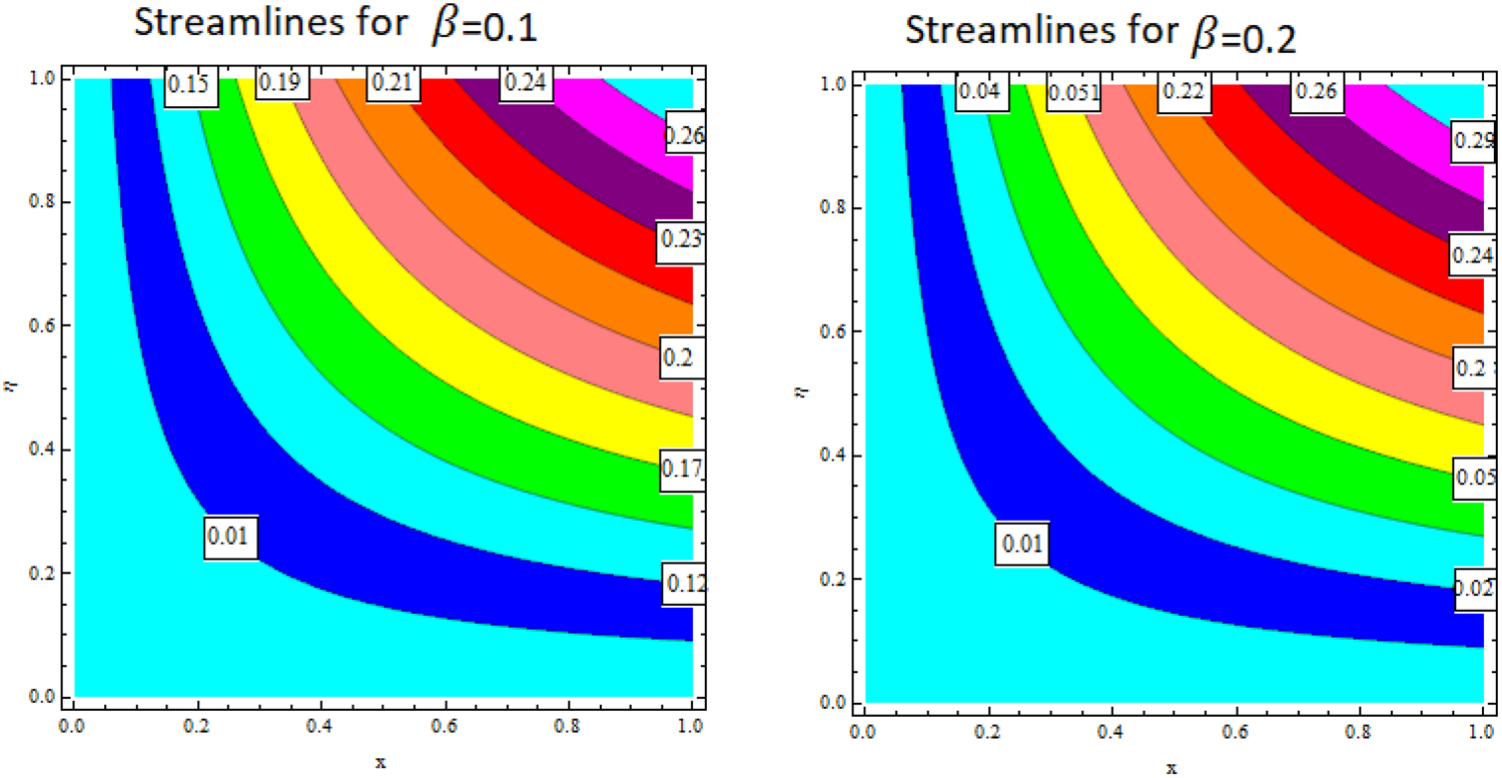
Streamlines for $g{^\prime}\left(\eta \right)$ using different values of $\beta$.

The relationship between the second-grade fluid factor and fluid velocity is shown in Fig. 22. This analysis depicts that the velocity curves enhance as the quantities of $\beta$ enhance. Figure 23 debates the influence of magnetic field $M$ on profile $g{^\prime}(\eta )$. It is detected that the velocity curves decrease as the magnetic strength is enhanced. This is owed to Lorentz force occurs in the existence of a magnetic field which slows down the profile $g{^\prime}(\eta )$. The outcome of the ${b}_{1}$ on the velocities $f{^\prime}(\eta )$ and $g{^\prime}(\eta )$ (x and y-direction) is reported in Figs. 24 and 25, respectively. When the velocity slip factor is increased, velocity curves decline. The influences of $M$ and $\beta$ on the velocity curves $f{^\prime}(\eta )$ are shown in Figs. 26 and 27 respectively. It is scrutinized that the flow rate decreases due to increase in magnetic factor while an increasing behavior is observed for $\beta$. Streamlines for the different values of $\beta$ is represented in Fig. 28. It is observed that the streamlines are increasing function of $\beta$. When the values of $\beta$ are increased the streamlines are also enhances.

To propose a truthfully feasible solution, it is significant to scrutinize the stability analysis of solutions when dual branches exist. Frequently, the first branch is indicated as a physical solution since it encounters far-field boundary conditions precisely, but this situation is validated to privilege which solution is stable without examining the stability implementation. The identified solution could be the second solution. Thus, stability analysis is performed to obtain the stability solutions. Therefore, the fundamental equations given in Eqs. (7)–(11) with Eq. (13) are made unsteady. The stability solution is obtained of the steady flow $f\left(\eta \right)={f}_{0}\left(\eta \right), g\left(\eta \right)={g}_{0}\left(\eta \right), \theta \left(\eta \right)={\theta }_{0}\left(\eta \right), and \phi \left(\eta \right)={\phi }_{0}\left(\eta \right)$ by expressing $f\left(\eta , \tau \right)={f}_{0}\left(\eta \right)+{e}^{-\gamma \tau }J\left(\eta ,\tau \right)$,$g\left(\eta , \tau \right)={g}_{0}\left(\eta \right)+{e}^{-\gamma \tau }F\left(\eta ,\tau \right)$,$\theta \left(\eta , \tau \right)={\theta }_{0}\left(\eta \right)+{e}^{-\gamma \tau }G\left(\eta ,\tau \right)$, $\phi \left(\eta \right)={\phi }_{0}\left(\eta \right)+{e}^{-\gamma \tau }H\left(\eta ,\tau \right)$ which satisfy the boundary flow problem as proposed by Merkin^31^ and Wiedman et al.^32^. Here, $\gamma$ is an undetermined eigenvalues and $J\left(\eta ,\tau \right)$,$F\left(\eta ,\tau \right)$, $G\left(\eta ,\tau \right)$ and $H\left(\eta ,\tau \right)$ are relatively small to ${f}_{0}\left(\eta \right),$ ${\theta }_{0}\left(\eta \right),$ ${g}_{0}\left(\eta \right)$, and ${\phi }_{0}\left(\eta \right)$. After simplification, the resultant equations are solved numerically by bvp4c MATLAB's software. During this investigation, we elucidated Eqs. (30)–(33) with the assistance of a bvp4c approach to obtain the values of $\gamma$. Signs of $\gamma$ contribution to suggest a stable branch. The solution is the stable for the positive eigenvalues $\gamma$ and the negative sign of $\gamma$ indicates the unstable. The stable solution is represented as first solution or first branch and the unstable is termed as the second solution or second branch as illustrate in Table 2.

**Table 2:** Smallest eigenvalues $\gamma$ for selected values of $\mathrm{Pr}$ and ${\lambda }_{1}$.

$Pr$	${\lambda }_{1}$	First solution $(\gamma )$	Second solution $(\gamma )$
0.5	− 1.5125	0.0016	− 0.0015
	− 1.512	0.0021	− 0.0020
1.0	− 1.51	0.0480	− 0.0370
	− 1.4134	0.0225	− 0.0224
2.0	− 1.41	0.0252	− 0.0241
	− 1.4	0.0057	− 0.0045

## Conclusions

Three-dimensional incompressible flow over the slendering stretching/shrinking sheet is examined in the existence of magnetic field, chemical reaction, thermal radiation, and nonlinear heat generation/absorption. The nonlinear PDEs are transformed into nonlinear dimensionless ODEs and computed numerically via bvp4c in Matlab software. Dual branches (solutions) are observed for some parameters like magnetic field and stretching/shrinking factors. The stability investigation is implemented in order to examine the reliable solution. The lowest eigenvalues are determined for this purpose. The positive sign of eigenvalues signifies the stable solution while the negative sign indicates the unstable solutions. The magnitude of the local Nusselt number is compared with the previous work to confirm the gained numerical results which show a good agreement. The significant consequences of the existing analysis are:

1. Two sets of branches are observed specifically dual solutions and no solution
2. No dual branches is existed for the parameters ${M}_{c}$ and ${\lambda }_{1c}$
3. The heat rate enhances as the thermophorsis factor is declined
4. The profile $\upphi (\upeta )$ enhances as the quantity of chemical reaction is improved
5. It is witnessed that the profile $g{\prime}(0)$ is decelerated as the factor $\beta$ is augmented
6. It is detected that the concentration improves for the increasing quantities of $L$ while opposite influence is portrayed on temperature
7. It is revealed that due to the increasing magnitude of $\delta$, the concentration curve improves in the channel.

## Data Availability

The datasets used and/or analysed during the current study available from the corresponding author on reasonable request.

## List of symbols

$u,v,w$: Velocities in $x,y$ and $z$ directions $\left({{\mathrm{ms}}^{-1}}^{-1}\right)$
$$T$$: Fluid Temperature $$(\mathrm{K})$$
$$C$$: Fluid Concentration $$\left(\mathrm{kg}/{\mathrm{m}}^{3}\right)$$
$$f\mathrm{^{\prime}}$$: Dimensionless Velocity
${g}^{\mathrm{^{\prime}}}$: Non-dimensional transverse velocity
$c$: Parameter associated with stretching
${b}_{1}^{*}$: Velocity slip parameter
${b}_{2}^{*}$: Thermal jump parameter
${b}_{3}^{*}$: Concentration jump parameter
$n$: Power Index parameter
$$a$$: Constant
${T}_{\infty }$: Free stream temperature $(\mathrm{K})$
${C}_{\infty }$: Free stream concentration (mol m^−^^1^)
$A$: Coefficient associated with a stretching sheet
$${Q}_{0}$$: Heat source $$\left(W {m}^{-3} {K}^{-1}\right)$$
$${D}_{T}$$: Thermophoresis coefficient
${D}_{B}$: Brownian diffusion coefficient $(\mathrm{kg}/\mathrm{ms})$
$${K}^{*}$$: Reaction rate
$$Nt$$: Thermophoresis parameter
$\mathrm{Nb}$: Brownian motion parameter
$\beta$: Second-grade fluid parameter
$$M$$: Magnetic parameter
$Q$: Heat generation parameter
$$R$$: Radiation parameter
$\delta$: Chemical reaction parameter
${b}_{1}$: Dimensionless velocity slip parameter
${b}_{2}$: Dimensionless thermal jump parameter
${b}_{3}$: Dimensionless concentration jump parameter
Sc: Schmidt number
$$\mathrm{Pr}$$: Prandtl number
$$\mathrm{Re}$$: Reynolds numbers
${f}_{1}$: Maxwell reflection coefficient
${\zeta }_{1}$: Mean free path
$${\alpha }_{1}$$: Stress moduli
$$\sigma$$: Electrical conductivity
$b$: Thermal accommodation coefficient
$m$: Concentration accommodation coefficient
$\lambda$: Wall thickness parameter
${\sigma }^{*}$: Stefan–Boltzmann constant $\left(W\, {m}^{-2}\,{K}^{-4}\right)$
$$\gamma$$: Adiabatic index
$$\tau$$: Heat capacitance
$$v$$: Kinematics viscosity $$\left({m}^{2} {s}^{-1}\right)$$
$$\rho$$: Fluid density $$\left({\mathrm{kg m}}^{-3}\right)$$
$$\mu$$: Dynamic viscosity
$$\theta$$: Dimensionless temperature
$$\phi$$: Dimensionless concentration
